# Characterization and selection of endophytic actinobacteria for growth and disease management of Tea (*Camellia sinensis* L.)

**DOI:** 10.3389/fpls.2022.989794

**Published:** 2022-11-09

**Authors:** Shabiha Nudrat Hazarika, Kangkon Saikia, Debajit Thakur

**Affiliations:** ^1^ Microbial Biotechnology Laboratory, Life Sciences Division, Institute of Advanced Study in Science and Technology, Guwahati, India; ^2^ Department of Molecular Biology and Biotechnology, Cotton University, Guwahati, India; ^3^ Bioinformatics Infrastructure Facility, Institute of Advanced Study in Science and Technology, Guwahati, India

**Keywords:** endophytic actinobacteria, diversity, plant growth promotion, antagonist, seed biopriming, biofilm formation

## Abstract

Endophytic microbes are vital for nutrient solubilization and uptake, growth, and survival of plants. Here, 88 endophytic actinobacteria (EnA) associated with five tea clones were isolated, assessed for their diversity, plant growth promoting (PGP), and biocontrol traits, and then used as an inoculant for PGP and disease control in host and non-host plants. Polyphasic methods, including phenotypic and genotypic characteristics led to their identification as *Streptomyces, Microbacterium, Curtobacterium, Janibacter, Rhodococcus, Nocardia, Gordonia, Nocardiopsis*, and *Kribbella.* Out of 88 isolates, 35 (39.77%) showed antagonistic activity *in vitro* against major fungal pathogens, viz. *Fusarium oxysporum, Rhizoctonia solani, Exobasidium vexans, Poria hypobrunnea*, *Phellinus lamaensis, and Nigrospora sphaerica*. Regarding PGP activities, the percentage of isolates that produced indole acetic acid, siderophore, and ammonia, as well as P-solubilisation and nitrogen fixation, were 67.05, 75, 80.68, 27.27, 57.95, respectively. A total of 51 and 42 isolates showed chitinase and 1-aminocyclopropane-1-carboxylic acid deaminase activity, respectively. Further, two potent *Streptomyces* strains KA12 and MA34, selected based on the bonitur scale, were screened for biofilm formation ability and tested *in vivo* under nursery conditions. Confocal laser scanning microscopy and the crystal violet staining technique revealed that these *Streptomyces* strains can form biofilms, indicating the potential for plant colonization. In the nursery experiment, they significantly enhanced the shoot and root biomass, shoot and root length, and leaf number in host tea plants. Additionally, treatment of tomato seeds by KA12 suppressed the growth of fungal pathogen *Fusarium oxysporum*, increased seed germination, and improved root architecture, demonstrating its ability to be used as a seed biopriming agent. Our results confirm the potential of tea endophytic actinobacterial strains with multifarious beneficial traits to enhance plant growth and suppress fungal pathogens, which may be used as bioinoculant for sustainable agriculture.

## 1 Introduction

Actinobacteria are widespread in natural and artificial environments. They have adapted to diverse ecological habitats and are mostly soil dwellers or saprophytes, and some exist in mutual or antagonistic relationships with plants or animals (Aamir et al., 2020). In recent times due to the versatility of this bacterial domain, new possibilities for novel metabolites have been opened up, such as novel drugs for antimicrobial or antineoplastic resistance, modest means for the maintenance of ecological balance, and eco-friendly agricultural practices (Barka et al., 2016; Azman et al., 2017; Shrivastava and Kumar, 2018). According to reports, 50% of the natural products are from actinobacteria out of 22000 natural products to be of microbial origin. But only a limited quantity is used in human medication and agriculture (Berdy, 2012); thus, to meet the escalating demand for such compounds and to broaden the repertory of bioactive compounds, there is an urgent need to explore actinobacteria further.

Conventionally soil actinobacteria have been the focus of intense research but lately, endophytic actinobacteria inhabiting as symbionts internally have been recognized as a prospective source to produce novel metabolites that may be of agricultural, environmental, and industrial importance (Strobel and Daisy, 2003; Qin et al., 2011). Studies unveiled that almost all plants foster endophytes which occupy unique biological niches and colonize almost all plant tissues (Strobel and Daisy, 2003; Stepniewska and Kuzniar, 2013). Endophytes are plant-associated microorganisms residing intra or inter-cellularly within plant tissues, mainly with a symbiotic relationship. These microbes play substantial roles in ameliorating plant growth and improving tolerance for abiotic and biotic stresses, and in turn, they benefit from shelter and nutrients (Hazarika and Thakur, 2020). Endophytic actinobacteria are reported to facilitate plant growth by supplying essential nutrients to plants through various mechanisms, including phosphate solubilization (Borah and Thakur, 2020), production of phytohormones like indole acetic acid (IAA) that promote both cell division and elongation of roots (Spaepen and Vanderleyden, 2011) biosynthesis of siderophore (Cruz-Morales et al., 2017), and also shield the plant by the production of bioactive antimicrobial metabolites to inhibit the growth of plant pathogenic microorganisms (Singh and Dubey, 2015). They also help the plant adapt to harsher environmental conditions by producing stress alleviating enzyme 1- aminocyclopropane-1- carboxylic acid deaminase (ACCD) (Glick, 2012).

Of the nearly 351,000 plant species (Pimm, 2020) that exist on earth, only a few of these plants are studied, such as cereal crops (Tian et al., 2007; Jog et al., 2014), solanaceous crops (Achari and Ramesh, 2014; Hassan, 2017), and legumes (Le et al., 2015) relative to their endophytic biology. Consequently, an opportunity to search for new endophytic actinobacteria among myriads of plants in different ecosystems is tremendous. Thus, in our current study, we have selected Tea (*Camellia sinensis* L. Kuntze) as it has not been thoroughly explored, despite being considered therapeutic. Tea possesses anti-oxidant, anti-inflammatory, antimicrobial, and anti-cancer properties. It helps to boost mental health, improve bone health, prevent heart diseases, fight obesity, and control diabetes (McKay and Blumberg, 2002; da Silva Pinto, 2013).

Tea plants have abundant endophytic bacteria and fungi (Xie et al., 2020). But there have only been a few studies on tea plant endophytes, and these studies have mainly concentrated on endophytic fungi and their role in plant disease resistance. For example, endophytic fungi *Aspergillus niger* and *Penicillium sclerotiorum* isolated from root, stem, and leaves of tea plants collected from Assam showed plant growth promoting traits (Nath et al., 2015). Some other endophytic fungi, *Colletotrichum gloeosporioides*, inhibited the growth of tea phytopathogens *Pestalotiopsis theae* and *Colletotrichum camelliae* (Rabha et al., 2014). Tea endophytic bacteria viz. *Pseudomonas* sp., *Stenotrophomonas* sp., *Bacillus* sp., and *Lysinibacillus* sp. are also reported showing potential PGP traits (Borah et al., 2019; Hazarika et al., 2021). Endophytic bacteria strain *Bacillus subtilis* TL2 showed significant inhibition of mycelial development of tea pathogens *Gloeosporium theae-sinensis, Phyllosticta gemmiphliae, Pestallozzia theae*, and *Neocapnodium theae* (Xie et al., 2020). A study reported 46 endophytic actinobacteria isolated from 15 tea cultivars collected in Fujian province, China, with antibacterial, antifungal, and plant-growth-promoting properties (Shan et al., 2018). Another study isolated 16 and 28 endophytic actinobacteria with antimicrobial properties from two tea cultivars, Zijuan and Yunkang-10, respectively (Wei et al., 2018). These findings show the widespread potential of the endophytic community associated with tea. Nevertheless, the diversity and the plant beneficial traits of endophytic actinobacteria within tea vegetative clones of Northeastern India have not been thoroughly explored.

India is one of the world’s largest tea producers, especially in North-Eastern India, with humid climatic conditions, hilly terrain, and fertile soil, contributes to the growth of tea leaves, among which Assam tea is known worldwide (Borah et al., 2022). In this region, tea plants are susceptible to environmental stresses such as dry spells, waterlogging (Dutta, 2014), and various fungal diseases viz. blister blight, grey blight, root rot, branch canker, necessitating the use of chemical fertilizers, pesticides, and other nutrients to boost crop productivity (Barthakur, 2011; Barman et al., 2020; Pandey et al., 2021). Such agricultural practices have adversely affected soil health, leading to a degeneration of soil fertility and microbial diversity (Lin et al., 2019; Pandey et al., 2021). They have also rendered pests and insects resistant to these chemicals, and bioaccumulation of these chemicals is invoking severe health concerns in humans (Roy et al., 2020). These methods have been considered to be non-feasible, and thus, there is a prerequisite for developing and implementing viable alternatives. Besides tea plants, agricultural crops are also polluted by agrochemicals, posing health risks to humans and the environment (Aktar et al., 2009). Thus, finding an effective eco-friendly alternative way to remove pesticide residues and increase food security has become critical. As such, despite the fact that all endophytes have a degree of host specificity and a more symbiotic interaction with the host (Khare et al., 2018), we investigated the compatibility of endophytic actinobacteria isolated from tea in colonizing tomato plants used as a test plant to accelerate growth or suppress disease in non-host plants by seed biopriming techniques (seed treatment using beneficial biological agents to stimulate seed germination, development of the plant, and disease resistance). Although we have found reports where tea endophytes significantly promote wheat seed germination and seedling growth (Xie et al., 2020), there is very little information available regarding the role of tea endophytic actinobacteria in accelerating the growth or suppressing disease in non-host plants.

Thus, this study aims to isolate and characterize endophytic actinobacteria from tea clones of Northeastern India and investigate plant growth promoting traits and antifungal activities against fungal phytopathogens. This study will further establish a meaningful basis for developing new bio formulations for agricultural applications, mainly to enhance tea plants and reduce the usage of synthetic chemicals.

## 2 Materials and methods

### 2.1 Sample collection

Tocklai vegetative (TV) clones TV1, TV9, TV22, TV25, and Teenali 17 were explored for the habitation of probable endophytic actinobacteria. These tea clones were selected based on their economic value. These TV clones were vegetatively propagated by Tocklai Tea Research Institute (TTRI), Jorhat, Assam, India, and released to the industry for commercial cultivation, especially in Northeast India. These clones were identified manually with the help of experts in the tea industry. Healthy plant parts (leaf and roots) from three-year-old tea bush were collected from Kopati Tea estate, Assam, India (26.5875°N, 92.2507°E) and MEG Tea estate, Meghalaya, India (25.741729° N, 91.889094° E) by random sampling method (**Table 1**). The samples were collected aseptically in zip lock bags and brought to the laboratory in an ice box, and further processed for isolation within one month.

**Table 1:** Sample collection.

Sl. No.	Collected samples			Tissue Origin	Sampling site and GIS location
	Tea Clones	Jat/Population/Parents	Clone type		
**1**	TV1	Assam-China (Cinnamara)	Standard	Leaf, Root	Kopati Tea estate, Darrang, Assam, India (26.5875° N, 92.2507° E); Meg Tea, Tea Development Centre, Umsning, Meghalaya, India (25.741729° N, 91.889094° E)
**2**	TV9	Burma (Cambod)	Standard	Leaf, Root
**3**	TV22	Cambod type (Indo-China)	Yield	Leaf, Root
**4**	Teenali 17	Cambod type	Estate	Leaf, Root
**5**	TV25	Cambod type: Ayapathar D × Ayapathar A (DA/4)	Yield	Leaf, Root

### 2.2 Isolation of endophytic actinobacteria

Putative endophytic actinobacteria was isolated by the method described in our previous study (Hazarika et al., 2021). Briefly, surface-sterilized plant parts were macerated using mortar and pestle. Tissue extracts were then serially diluted in 0.9% sodium chloride and plated in selective media, namely Actinomycetes Isolation Agar, Streptomyces agar, ‘King’s B agar, International Streptomyces Project (ISP) series agar ISP7, ‘Pikovskaya’s agar, Nutrient agar with the addition of 75 µg L^-1^ of Amphotericin B and 2.5 µg L^-1^ of Rifampicin were used. Further, culture plates were incubated at 28 ± 2°C for 21 days.

### 2.3 Actinobacteria growth conditions and morphological characterization of EnA isolates

All the pure EnA isolates were grown on GLM agar plates containing 3 g L^-1^ each of malt extract and yeast extract, 5 g L^-1^ of Peptone, 10 ml L^-1^ of Glycerol, and 20 g L^-1^ of Agar, pH 7.4 at 28 ± 2°C for 10 days for initial phenotypic characterization and to obtain clonal isolates. The distinguished colonies were characterized based on typical actinobacterial phenotypic morphologies such as aerial and substrate mycelia, filamentous growth, dusty, rough, dry, and frequently pigmented colonies. These morphological characteristics of the EnA isolates were accounted based on the International Streptomyces Project (Shirling and Gottlieb, 1966).

### 2.4 Taxonomic relationship between the actinobacteria isolates

16S rRNA gene sequences were analyzed to determine the taxonomic relationships among the EnA isolates. Genomic DNA was extracted using Nucleopore Fungus/bacteria kit (Genetix, India). The concentration and purity of the eluted DNA were quantified by a Nanodrop spectrophotometer (Thermo Fisher Scientific, United States). 16S rRNA gene was amplified using universal primers 27F and 1492R (Hazarika et al., 2021). PCR cycling conditions were: 5 min at 94°C, 30 s at 94°C, 30 s at 52°C, 1 min at 72°C, and 7 min at 72°C. The 16S rRNA gene sequence of each isolate was compared with the homologous sequence using the BLAST (Basic Local Alignment Search Tool) program of NCBI (National Center for Biotechnology Information) to identify the closest known taxa. The 16S rRNA gene sequences obtained were submitted to GenBank after chimera check Decipher 2.20 (Wright et al., 2012), and accession numbers were obtained. The evolutionary relationship between the isolates was studied by the maximum likelihood method using RAxML (Stamatakis, 2014). Finally, with 1000 steps of bootstrap analysis, a phylogenetic tree was constructed and visualized using iTol (Letunic and Bork, 2019).

### 2.5 Diversity analysis of endophytic actinobacteria

The species richness (S), species evenness (J), the Shannon diversity index (H’), and the Simpson diversity index (D or SDI) of the isolated endophytic actinobacteria were calculated and compared to literature (Shannon, 1948; Simpson, 1949; Pielou, 1966)

Diversity analysis (Shannon index) was calculated as:


$$H'=-\sum_{i=1}^{S}p_{i}Ln\left(p_{i}\right)$$

The dominance index (Simpson diversity index) was calculated as:


$$D=1-\left(\frac{\sum n\left(n-1\right)}{N\left(N-1\right)}\right)$$

Species evenness was calculated as:


$$J={\frac{H'}{H'}}_{max}$$

where, *H*'_*max*_=*lnS* The Pi = ni/N, the proportion of individuals found in the *i*th species; ni is the number of the species i in the sample; S is the number of species (species richness); *Ln* is the natural log; N is the total number of individuals of all species; n is the number of individuals of a single species

### 2.6 Determination of plant-growth-promoting activities

#### 2.6.1 Phosphate solubilization

The EnA isolates were evaluated for their ability to solubilize inorganic phosphate by qualitative and quantitative methods. Actinobacteria isolates were assayed qualitatively by plate assay using ‘Pikovskaya’s medium at 28 ± 2°C for 10 days. Phosphate (P) solubilization was indicated by the appearance of clear or halo zones around colonies. Further, quantitative estimation was done in NBRIP (National Botanical Research ‘Institute’s Phosphate Growth) medium at 28 ± 2°C for 10 days. The incubated cultures were harvested, and the ammonium molybdate method determined phosphorus in the supernatant (Fiske and Subbarow, 1925).

#### 2.6.2 Indole acetic acid production

Indole acetic acid (IAA) screening and quantification were determined colorimetrically in terms of IAA equivalents using the Salkowski reagent method in the presence of L-tryptophan (L-TRP) (Gang et al., 2019). The pre-cultures of EnA were grown in GLM liquid medium at 28 ± 2°C at 180 rpm without tryptophan. To quantify the IAA produced by EnA isolates, the development of pink coloration was recorded at 535 nm, as described previously by (Gordon and Weber, 1951).

#### 2.6.3 Nitrogen fixation and ammonia production

For the selection of diazotrophic actinobacteria Nitrogen-free (NF) medium was used. The isolates were spot inoculated on NF media and based on the growth at 28 ± 2°C for 10 days, the nitrogen-fixing abilities were determined (Kifle and Laing, 2015).

All the isolates were screened *in vitro* for production of ammonia colorimetrically using Nessler’s reagent as described previously by (Yuen and Pollard, 1954; Singh et al., 2014).

#### 2.6.4 ACC deaminase production

ACC deaminase (ACCD) activity was determined qualitatively by growing isolates in Dworkin and Foster (DF) salt medium (Dworkin and Foster, 1958) supplemented with 3 mM ACC as the sole nitrogen source. The growth of EnA was monitored for 10 days at 28 ± 2°C, and ACC deaminase producers were recognized (Glick et al., 2007).

#### 2.6.5 Siderophore production

Siderophore production was determined by using Chrome Azurol S (CAS) shuttle assay (Schwyn and Neilands, 1987; Alexander and Zuberer, 1991). The resulted change in color from blue to orange is measured colorimetrically at 630 nm. The percentage of siderophore production units is estimated as described (Payne, 1937).


$$\text{Siderophore production units }\left(\text{psu}\right)=\;\frac{\left(Ar-As\right)}{Ar}\times 100$$

where *Ar*, *As* is the absorbance of the reference and sample respectively at 630 nm

### 2.7 Evaluation of biocontrol activity

Endophytic actinobacterial isolates were screened *in vitro* for the growth inhibition of phytopathogenic fungi, namely *Exobasidium vexans* (ITCC 938), *Poria hypobrunnea* (ITCC 4141), *Phellinus lamaensis* (ITCC 292), *Fusarium oxysporum* (MTCC 284), *Rhizoctonia solani* (MTCC 4633), *Nigrospora sphaerica* (KJ767520) which were collected from Indian Type Culture Collection (ITCC), IARI, New Delhi and Microbial Type Culture Collection (MTCC), IMTECH, Chandigarh and maintained in laboratory condition for experimental use. The pure culture of different pathogenic fungi was maintained in potato dextrose agar plates at 4°C, as glycerol stock at -20°C for experimental use, and as glycerol stock at -80°C for long storage. After a period of 3-4 months, the cultures are sub-cultured to maintain their metabolic activities. To determine the inhibition ability of the EnA isolates, an experiment was done in GLM agar media using the conventional spot inoculation method and incubated for 10 days at 28 ± 2°C. The five-day-old culture of fungal test pathogens was spread on plates with EnA isolates. Plates with only the target pathogen were used as control. Each experiment was conducted in three replicates. Plates were incubated for 7 days at 28 ± 2°C. The antifungal effect was considered by correlating the diameter of fungal mycelial on test plates with control plates and estimating the fungal growth inhibition (GI%). GI % was computed using the formula C-T/C×100, where C and T are the mycelial diameter on plates with control and EnA isolates, respectively (Sgroy et al., 2009).

### 2.8 Chitinase enzyme production

Extracellular chitinase production was determined by using chitin agar plate consisting of (per liter) 0.5 g Yeast extract, 1 g (NH_4_)_2_SO_4_, 0.3 g MgSO_4_.6H_2_O, 1.36 g KH_2_PO_4_, 2 g agar amended with 1.5 g colloidal chitin. The screening was done by spot inoculation and incubation of agar plates at 28 ± 2°C for 10 days. Chitinase production was confirmed by observing the clear or halo zones developed in the vicinity of the colonies (Roberts and Selitrennikoff, 1988).

### 2.9 Biopriming role of endophytic actinobacteria KA12 for enhancing seed germination and disease management

The tomato (*Solanum lycopersicum*) seeds were surface sterilized with 2% sodium hypochlorite (NaOCl) for 10 min, and to remove traces of the disinfectant, the seeds were rinsed with sterile water for 5 min. The surface-sterilized seeds were then allowed to dry in a sterile environment. Surface sterilization was followed by seed bacterization by the method previously described (Niknam and Dhawan, 2003). In brief, fresh cultures of KA12 actinobacteria were prepared in GLM broth. Before seed bacterization, seeds were coated with 0.1% carboxymethylcellulose (CMC), followed by the addition of actinobacterial suspension (10^8^ cells mL^-1^) as seed drench. For each experiment, a ratio of 10 ml per 30 seeds was applied and kept for 30 min, followed by air drying the seeds for 3 h in a sterile condition. The seed bacterization was validated by serial dilution and spread plating method.

To determine the seed biopriming effect of KA12, tomato seeds were treated with 100 μl of freshly cultured *F. oxysporum* spore suspension and incubated for 24 h. Seeds soaked under the same conditions in 10 ml of sterile distilled water with and without fungal inoculation served as a positive and negative control, respectively. Additionally, seeds incubated with GLM broth and 0.1% CMC served as the negative control. The seeds were then aseptically placed onto 3% w/v water agar plates moistened with sterile filter paper. Plates were incubated under a 16 h light/8 h dark cycle for up to 14 days at 28 ± 2°C. The degree of disease symptoms and inhibition of *F. oxysporum* on tomato seeds were determined by visual inspection after 14 days of inoculation. In addition, the seed germination rate per plate was determined, and the root architecture was studied in a stereomicroscope (Stemi 508, Carl Zeiss Microscope.). Each seed was assigned a disease severity score from 0 to 5, with 0 indicating no symptoms, 1 indicating less than 10% infection, 2 indicating 10% infection, 3 indicating 25% infection, 4 indicating 50% infection, and 5 indicating more than 75% infection. Further, the rate of seed germination was also scored, with 0 indicating no germination, 1 indicating germinated seed with seed infection, and 2 indicating germinated seed with no infection. The overall score in each treatment was calculated by multiplying the score awarded to each treatment by the number of seeds (n). Each experiment included three replications and 15 seeds per treatment (n).

### 2.10 Effect of potent actinobacterial isolates on tea plant growth

To evaluate plant growth in tea ‘clones’ an experiment was carried out in sleeves under the effect of actinobacterial inoculum. Three healthy tea clones of 3-4 months old, namely TV22, TV26, and TV9, were collected from Bateli Tea estate, Assam. The sleeves contained sandy loam soil with the succeeding characteristics: 50 - 70% sand greater than 0.2 mm, 0 - 50% silt 0.002 - 0.2 mm, and 15–20% clay less than 0.002 mm of good tilth with an ideal pH of 4.5 (Hazarika et al., 2021). For inoculum preparation, two potent actinobacterial strains KA12 (*Streptomyces* sp., GenBank Accession number MN483270.1) and MA34 (*Streptomyces* sp. GenBank Accession number OK483112.1), were cultured in GLM broth at 28 ± 2°C for 10 days as optimized. The obtained biomass was harvested by centrifugation at 7000 rev min^-1^ for 10 min, washed thrice with 1X phosphate-buffered saline (PBS) buffer, and adjusted to 10^8^ CFU mL^-1^. In nursery conditions (average temperature of 30°C, average soil moisture of 5-6 RH, light intensity of 250 μmol m^-2^ s^-1^), the tea clones were treated for 15 days for 6 months by the soil drenching method. Three treatments were conducted on the tea saplings, including T1: KA12 inoculation, T2: MA34 inoculation, T3: consortia of KA12 and MA34, and control, without actinobacterial inoculum. Each experiment included three replicates. Vegetative parameters of plants, including the fresh and dry weight of shoot and root, shoot and root length, and the number of leaves, were recorded and compared to control.

### 2.11 Test for root colonization

To determine the root colonization ability of the potent endophytic isolates to *C. sinensis* and *S. lycopersicum* roots, rhizospheric soil was carefully detached and washed with sterile distilled water to remove any adhered soil particles. To release the actinobacteria into the buffer, roots of 1 cm long uniform segments were cut and dipped into 5 ml of sterile 1X PBS buffer, pH 7.4, and vortexed for 5-6 times to release the actinobacteria into the buffer. To evaluate the population of actinobacteria, solutions of actinobacterial suspensions were spread on GLM agar plates and incubated for 48-72 h at 28 ± 2°C, followed by counting of CFU. The primary indication of their competence to endophytically colonize the roots was determined by the presence of viable actinobacteria (Passari et al., 2016b; Singh and Jha, 2016). *In vitro* biofilm formation by actinobacterial isolates was also studied by Confocal Laser Scanning Microscope (CLSM) (Hazarika et al., 2021).

### 2.12 Chlorophyll estimation

The chlorophyll content in tea leaves was determined as described by (Hiscox and Israelstam, 1979; Hazarika et al., 2021). 100 mg of mature tea leaves were obtained from the treated and untreated tea clones for extraction of chlorophyll. The amount of chlorophyll was determined at 645 nm and 663 nm and calculated by using the following formula:


$$Chlorophyll\;a\;\left(mg/g\right)=12.7\;\left(A663\right)-2.69\;\left(A645\right)x\frac{V}{1000xW}$$

$$Chlorophyll\;b\;\left(mg/g\right)=22.9\;\left(A645\right)-4.68\;\left(A663\right)x\frac{V}{1000xW}$$

where A= absorbance at specific wavelengths; V= final volume of chlorophyll extract; W= fresh weight of tissue extracted.

### 2.13 Elemental analysis

Following the *in vivo* plant growth promotion experiment, the shoot elemental content of treated and untreated plants was evaluated. Sample digestion was carried out as outlined by AOAC method 935.13. For instance, 2.5 g dry matter of leaf were added to 30 ml of nitric acid (HNO_3_) in a digestion flask and refluxed for 30 min and continued till brown fumes disappeared. It was allowed to cool to room temperature and 10 ml of 70% perchloric acid (HClO_4_) was added and refluxed to make the solution clear. 50 ml of distilled water was then added and heated until effervescence was minimal. The final volume was adjusted to 100 ml using distilled water and filtered for further elemental analysis (AOAC, 2000; Almehdi et al., 2019). The phosphorus (P) content in the filtrates was determined by alkalimetric ammonium molybdophosphate method as described by AOAC official method 964.06 (AOAC, 2000). The other elemental components including Fe, Cu, Zn, Na, Ni, Mg, Mn, and Ca was assessed by Atomic Absorption Spectroscopy (AAS) (Model: AA7000, Shimadzu) as described by AOAC official method 975.03. CHNSO Analyzer (Model: FLASH*Smart*, Thermo Scientific) was used to determine the nitrogen (N) content in the shoots by automated method as described by AOAC official method 972.43 (AOAC, 2000).

### 2.12 Statistical analysis

All the experiments were performed in replicates of three and represented as mean ± standard deviation (SD). To study the significance level in the interrelationships among the treatments and vegetative parameters, the data obtained were statistically analyzed by two-way ANOVA (analysis of variance) in SPSS package 28.0. Variance in elemental analysis was determined by one-way ANOVA and *post hoc* test by Tukey’s highest significance difference. The significance of the effect was determined at p ≤ 0.05. In addition, fold change analysis in a log2 scale was established to study the up or down-regulation of parameters in the presence of treatments compared to the control. Further, multivariate principal component analysis (PCA) was administered on the datasets using (MATLAB R (2017a)). PCA analysis established a relationship among the samples to assess the influence of treatment on plant growth promotion.

## 3 Results

### 3.1 Isolation, identification, and diversity of endophytic actinobacteria

A total of 88 endophytic actinobacterial isolates of distinct colony morphologies were isolated from five different commercially important tea clones collected from the tea estates of Assam and Meghalaya (**Figure S1** and **Table S1**). Largest part of the endophytic actinobacteria isolates belonged to the roots (n = 63, 71.59%) followed by leaves (n = 25, 28.40%).

**Figure 1:**
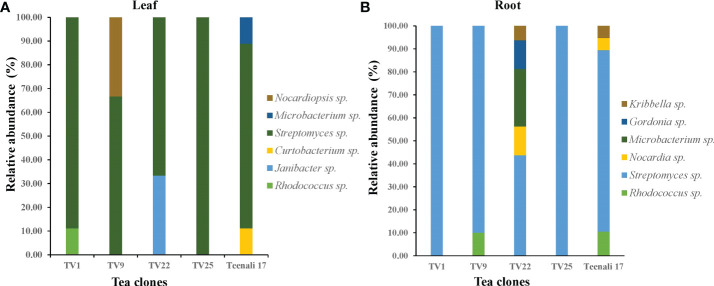
Relative abundance (in percentage) of endophytic actinobacterial genera procured from **(A)** leaf and **(B)** roots of five different tea clones (TV1, TV9, TV22, TV25, and Teenali 17).

**Figure 2:**
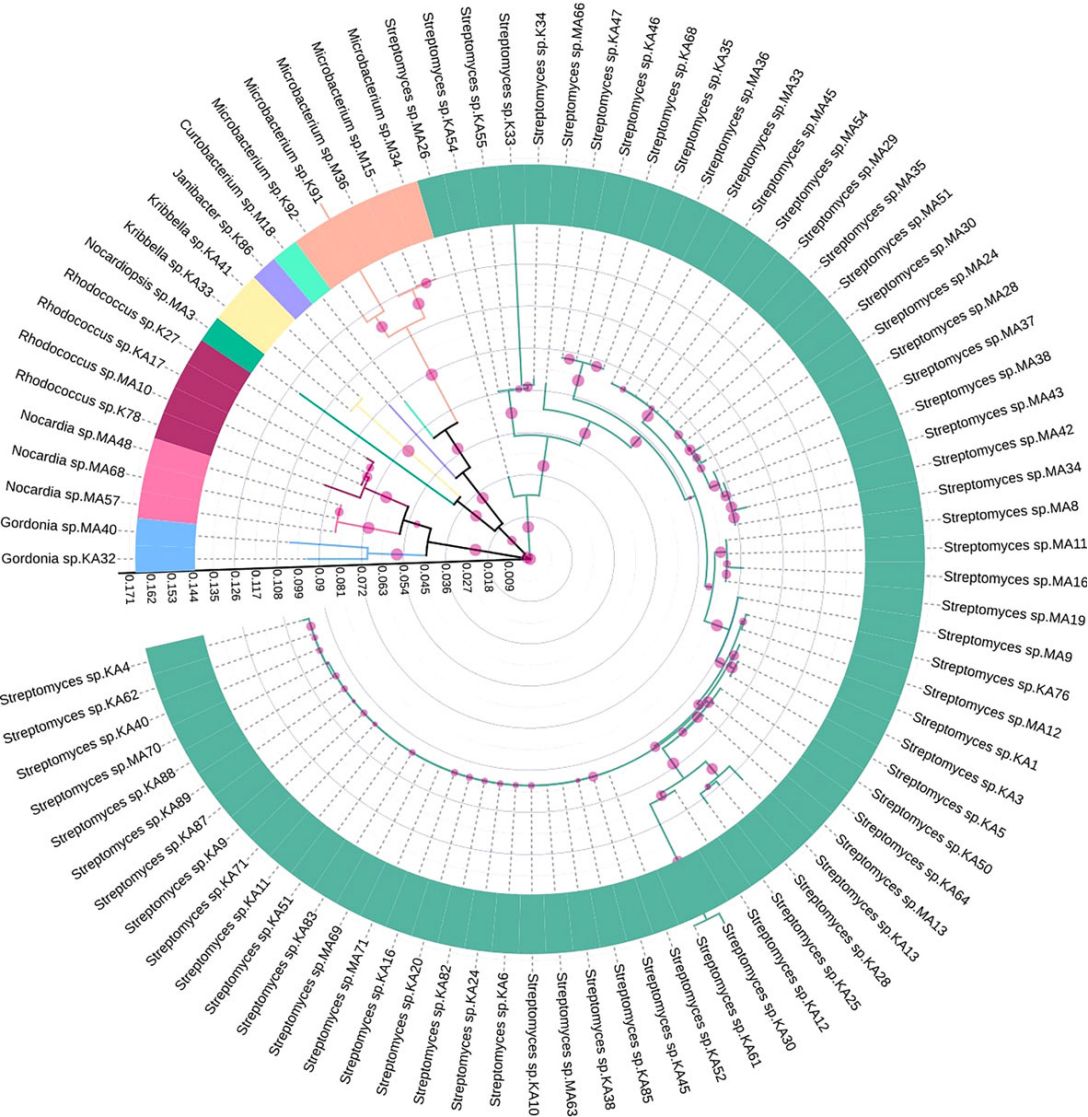
Maximum likelihood tree showing genetic diversity of different endophytic actinobacteria isolated from *Camellia sinensis* based on 16S rRNA gene sequences. Different genera were represented by individual colors. The scale bar represents the phylogenetic distance between taxa and clades. The circles with different sizes represent bootstrap values in respective branches.

Based on 16S rRNA gene sequencing results and analysis using the nucleotide BLAST tool of NCBI (**Table S2**), 88 actinobacterial isolates were divided into 9 genera. The respective genera abundance was different among the samples of tea clones TV1, TV9, TV22, TV25, and Teenali 17. Isolates belonging to genera *Streptomyces* was most abundant in all the tea clones irrespective of the plant parts (**Figure 1**). Taxonomy delineation revealed that all the strains belong to Phylum Actinobacteria, Class Actinobacteria, 5 orders, namely *Streptomycetales, Micrococcales, Mycobacteriales, Streptosporangiales, and Propionibacteriales*, 7 families namely *Streptomycetaceae*, *Microbacteriaceae, Intrasporangiaceae, Nocardiaceae, Gordoniaceae, Nocardiopsaceae, and Kribbellacea*, and 9 genera namely *Streptomyces, Microbacterium, Curtobacterium, Janibacter, Rhodococcus, Nocardia, Gordonia, Nocardiopsis*, and *Kribbella*. The evolutionary relationship between the isolates is shown in the phylogenetic tree constructed based on the maximum likelihood method (**Figure 2**).

Our study observed an average population size (µ) of 9.78. The species richness index (S) was found to be 9, and due to the reduced number of culturable isolated species, the diversity indexes were calculated for the overall samples. The Shannon diversity (H’), Simpson dominance (D), and evenness index (J) of the endophytic actinobacteria isolated from the tea clones were calculated. H’ was 0.934, which is based on randomness and represents a less diverse population. D was 0.623, which expressed overall dominance that considers the proportion of species. The uniformity of individual species in a community is determined by J, which ranges between 0 to 1(0 - no evenness and 1- complete evenness). The J index was 0.425, signifying a non-even distribution of genera among the isolated species.

### 3.3 Assessment of *in vitro* plant growth-promoting traits

**Figure 3:**
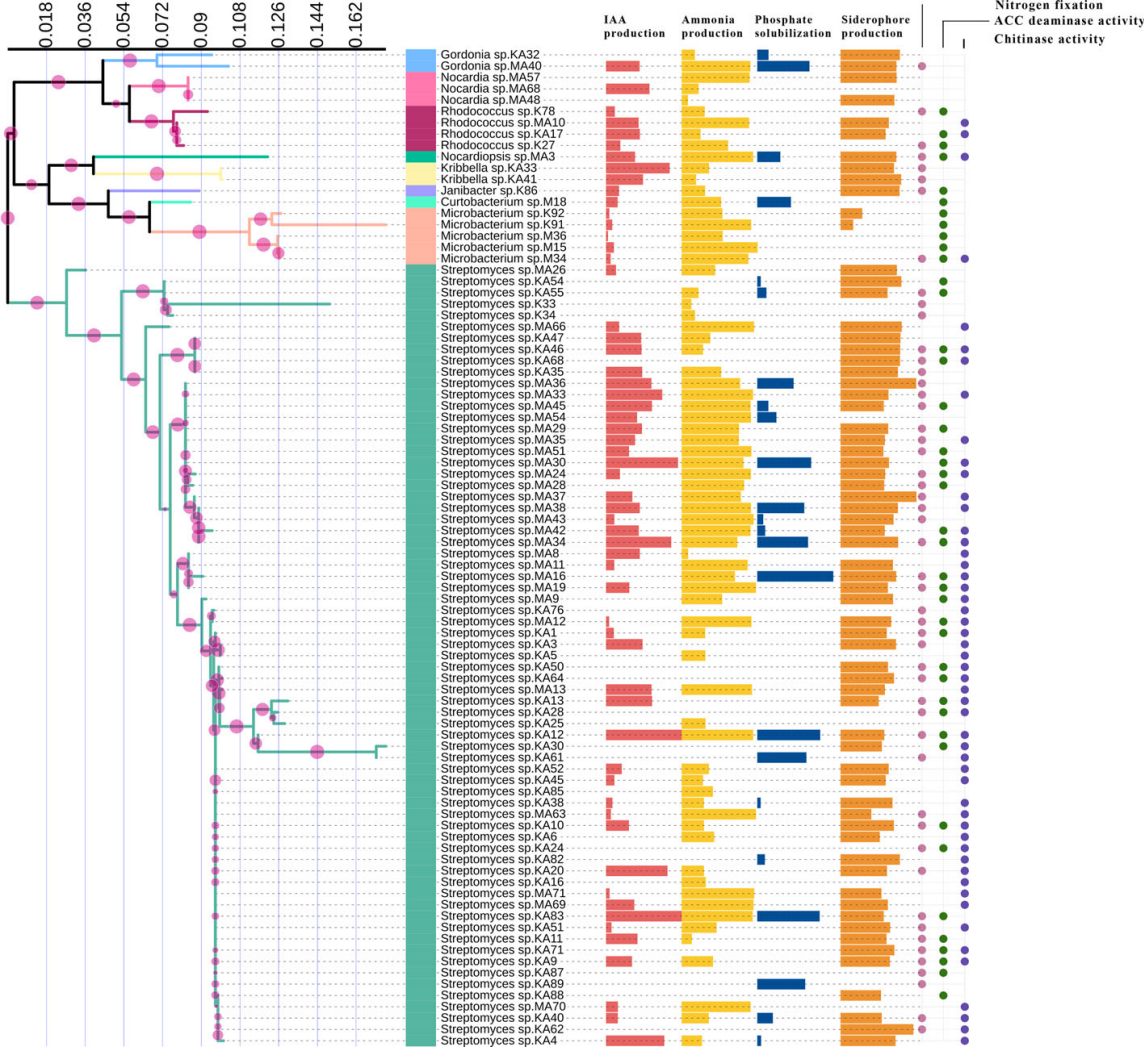
Comparison of PGP traits among different taxa of endophytic actinobacteria isolates. IAA production, ammonia production, phosphate solubilization, and siderophore production are represented by horizontal bars; circles represent the qualitative presence or absence of nitrogen fixation, ACC deaminase, and chitinase activity.

*In vitro* assessment of 88 endophytic actinobacterial isolates for the production of plant growth-promoting traits using qualitative and quantitative assays revealed that 59 (67.05%) isolates produced phytohormone IAA in the range 3.09 - 143 µg/ml, 24 (27.27%) isolates solubilized calcium phosphate available in the media in the range 2.48 - 62.19 µg/ml, 71 (80.68%) isolates produced ammonia in the range 0.39 - 4.92 µmol/ml, 66 (75%) isolates were found to be siderophore producers in the range 14.09- 86.33% SU. Nitrogen fixation was shown by 51 (57.95%) isolates, by their ability to grow in nitrogen-free media. 42 (47.73%) isolates produced ACC deaminase, an enzyme involved in abiotic stress alleviation, Maximum IAA of 143 µg/ml was produced by isolate KA12 (*Streptomyces* sp.), isolate MA16 (*Streptomyces* sp.) solubilized highest amount of phosphate of 62.19 µg/ml, maximum ammonia of 4.92 µmol/ml was produced by isolate M15 (*Microbacterium* sp.), isolate MA37 (*Streptomyces* sp.) produced maximum siderophore of 86.33% SU among the 88 isolates. Most isolates belonging to the genus *Streptomyces* were observed to fix nitrogen and produce ACC deaminase (**Table 2**). The capability of endophytic actinobacteria to ameliorate plant growth was also represented by a phylogenetic approach. Most of the positive strains were related to genus *Streptomyces*. The results obtained are illustrated in **Figure 3**. Richness in the PGP traits was consistently observed in the isolates KA12 (*Streptomyces* sp.), KA83 (*Streptomyces* sp.), MA3 (*Nocardiopsis* sp.), MA34 (*Streptomyces* sp.), and MA45 (*Streptomyces* sp.).

**Table 2:** *In vitro* plant growth-promoting and antifungal traits shown by endophytic actinobacterial isolates.

Sl. No.	Isolate Code	IAA production (µg mL^-1^) *	P solubilization (mg L^-1^) *	Ammonia production (µmol mL^-1^) *	Siderophore production (%) *	Nitrogen fixation	ACC deaminase activity	Inhibition of mycelia growth (%)					
								1	2	3	4	5	6
**1**	KA1	14.04 ± 0.65	ND	1.51 ± 0.06	52.40 ± 0.004	+	+	59.37 ± 1.1	55 ± 0.69	–	–	–	–
**2**	KA3	68.51 ± 0.29	ND	ND	62.93 ± 0.001	+	–	–	–	–	–	–	–
**3**	KA4	109.57 ± 0.36	2.8 ± 0.47	1.3 ± 0.06	62.44 ± 0.005	–	–	–	–	–	–	–	–
**4**	KA5	ND	ND	1.52 ± 0.01	ND	–	–	–	–	–	–	–	–
**5**	KA6	ND	ND	2.1 ± 0.04	44.42 ± 0.006	–	–	–	–	75 ± 0.47	–	–	–
**6**	KA9	48.35 ± 0.27	ND	2.02 ± 0.04	55.95 ± 0.005	+	+	71.875 ± 2.5	47.5 ± 1.5	–	–	–	–
**7**	KA10	42.56 ± 0.35	ND	1.43 ± 0.05	60.57 ± 0.004	+	+	–	65 ± 2.04	–	–	–	–
**8**	KA11	58.84 ± 0.34	ND	0.64 ± 0.02	52.16 ± 0.002	+	+	–	–	–	–	–	–
**9**	KA12	143 ± 0.35	51.44 ± 3.28	4.63 ± 0.07	49.60 ± 0.004	+	+	71.875 ± 0.42	55 ± 1.2	72.5 ± 1.6	42.86 ± 2.1	–	72 ± 1.03
**10**	KA13	86.42 ± 0.27	ND	ND	43.22 ± 0.004	+	+	–	57.5 ± 0.62	75 ± 1.1	–	–	80 ± 0.82
**11**	KA16	ND	ND	1.55 ± 0.04	ND	–	–	–	–	–	–	–	–
**12**	KA17	63.34 ± 0.044	ND	1.21 ± 0.07	51.11 ± 0.007	–	+	–	–	72.5 ± 1.3	–	–	–
**13**	KA20	115.57 ± 0.29	ND	1.43 ± 0.08	52.65 ± 0.003	+	–	78.125 ± 1.2	–	–	67.86 ± 1.13	–	–
**14**	KA24	ND	ND	0	ND	+	+	65.625 ± 0.45	–	–	–	–	–
**15**	KA25	ND	ND	1.53 ± 0.03	ND	–	–	–	–	–	–	–	–
**16**	KA28	ND	ND	ND	ND	+	+	–	–	–	–	–	–
**17**	KA30	ND	ND	ND	46.87 ± 0.003	–	+	–	–	–	–	–	–
**18**	KA32	ND	8.96 ± 0.72	0.84 ± 0.016	67.42 ± 0.002	–	–	–	–	–	–	–	–
**19**	KA33	119.58 ± 0.21	0	1.76 ± 0.11	60.71 ± 0.003	+	–	–	–	–	–	–	–
**20**	KA35	67.77 ± 0.17	0	2.55 ± 0.07	65.18 ± 0.020	+	–	–	–	–	–	–	–
**21**	KA38	11.59 ± 0.22	2.48 ± 0.09	1.42 ± 0.102	58.96 ± 0.004	–	–	–	–	–	–	–	–
**22**	KA40	21.54 ± 0.26	12.49 ± 0.38	1.74 ± 0.06	46.69 ± 0.002	+	–	–	–	–	–	–	–
**23**	KA41	69.22 ± 0.12	ND	0.92 ± 0.04	68.88 ± 0.003	+	–	–	–	–	–	–	–
**24**	KA45	15.17 ± 0.41	ND	1.36 ± 0.06	51.17 ± 0.001	–	–	–	–	–	–	–	–
**25**	KA46	66.49 ± 0.17	ND	1.37 ± 0.04	67.8 ± 0.003	+	+	68.75 ± 1.3	55 ± 2.06	–	–	–	–
**26**	KA47	65.79 ± 0.25	ND	1.84 ± 0.07	68.09 ± 0.005	–	–	–	–	–	–	–	–
**27**	KA50	ND	ND	0	53.88 ± 0.005	+	+	–	57.5 ± 1.14	–	–	–	–
**28**	KA51	9.72 ± 0.20	ND	2.25 ± 0.05	56.27 ± 0.003	+	–	–	–	–	–	–	–
**29**	KA52	29.11 ± 0.75	ND	1.75 ± 0.05	54.63 ± 0.005	–	–	–	–	–	–	–	–
**30**	KA54	ND	2.53 ± 0.74	ND	69.09 ± 0.004	–	+	–	–	–	–	–	–
**31**	KA55	ND	7.21 ± 0.54	1.07 ± 0.08	53.32 ± 0.001	+	+	–	–	–	–	–	–
**32**	KA61	ND	40.11 ± 1.1	ND	ND	+	–	–	–	–	–	–	–
**33**	KA62	ND	ND	ND	82.77 ± 0.002	+	–	65.625 ± 1.1	–	–	–	90 ± 1.15	–
**34**	KA64	ND	ND	ND	60.72 ± 0.001	+	+	62.5 ± 1.3	–	–	–	63.33 ± 1.3	–
**35**	KA68	ND	ND	ND	67.61 ± 0.005	+	+	–	45 ± 0.92	–	–	–	–
**36**	KA71	ND	ND	ND	61.16 ± 0.002	+	+	–	40 ± 1.1	–	–	–	–
**37**	KA76	ND	ND	ND	ND	+	–	–	–	–	–	–	–
**38**	KA82	ND	5.95 ± 0.92	ND	67.44 ± 0.002	–	–	–	–	–	–	–	–
**39**	KA83	142.75 ± 0.35	51 ± 3.28	4.60 ± 0.07	49.00 ± 0.004	+	+	43.75 ± 2.03	–	–	–	–	–
**40**	KA85	ND	ND	2.01 ± 0.03	ND	–	–	–	–	–	–	–	–
**41**	KA87	ND	ND	ND	ND	+	+	–	–	–	–	–	–
**42**	KA88	ND	ND	ND	45.87 ± 0.003	–	+	–	–	–	–	–	–
**43**	KA89	ND	39.11 ± 1.1	ND	0	+	–	–	–	–	–	–	–
**44**	MA3	54.21 ± 1.12	18.62 ± 2.01	4.63 ± 0.07	63.34 ± 0.010	+	+	–	–	–	–	–	40 ± 1.15
**45**	MA8	63.2 ± 1.32	0	0.40 ± 0.016	ND	–	–	–	–	–	–	–	–
**46**	MA9	ND	0	2.61 ± 0.26	59.64 ± 0.003	–	+	–	–	–	–	–	38 ± 2.04
**47**	MA10	61.02 ± 1.31	0	4.37 ± 0.09	54.80 ± 0.006	–	–	–	–	–	–	–	–
**48**	MA11	15.04 ± 0.35	0	4.27 ± 0.08	59.53 ± 0.016	–	–	–	–	–	–	–	–
**49**	MA12	5.28 ± 0.13	0	4.52 ± 0.07	57.46 ± 0.001	+	+	–	–	–	–	–	36 ± 1.42
**50**	MA13	85.57 ± 1.36	0	4.55 ± 0.14	50.31 ± 0.004	–	–	–	–	–	–	–	–
**51**	MA16	ND	62.19 ± 3.94	3.44 ± 0.02	63.21 ± 0.000	+	+	75 ± 0.89	–	–	–	–	34 ± 1.16
**52**	MA19	43.36 ± 1.10	ND	4.81 ± 0.03	53.68 ± 0.002	+	+	87.5 ± 0.75	–	–	–	–	–
**53**	MA24	25.57 ± 0.144	ND	4.48 ± 0.11	50.59 ± 0.003	+	+	81.25 ± 1.3	–	–	–	–	36 ± 1.32
**54**	MA26	18.3 ± 0.29	ND	2.16 ± 0.04	64.03 ± 0.001	–	–	–	–	–	–	–	–
**55**	MA28	ND	ND	4.04 ± 0.03	49.50 ± 0.013	+	+	62.5 ± 1.17	–	–	–	–	–
**56**	MA29	67.32 ± 0.32	ND	3.71 ± 0.03	54.11 ± 0.014	+	+	62.5 ± 0.95	–	–	–	–	26 ± 0.87
**57**	MA30	135.43 ± 2.6	44.02 ± 0.14	4.00 ± 0.13	54.93 ± 0.022	–	+	62.5 ± 1.16	–	–	–	–	24 ± 0.96
**58**	MA33	105.65 ± 1.18	ND	4.62 ± 0.11	54.40 ± 0.002	+	–	–	–	–	–	–	34 ± 0.85
**59**	MA34	122.64 ± 0.14	41.45 ± 3.28	3.60 ± 0.07	65.48 ± 0.004	+	+	71.875 ± 0.51	–	–	–	42 ± 0.59	42 ± 1.14
**60**	MA35	54.1 ± 1.24	ND	3.70 ± 0.12	50.23 ± 0.002	+	–	–	–	–	–	–	32 ± 0.95
**61**	MA36	85.24 ± 2.05	29.56 ± 0.98	3.78 ± 0.004	86.02 ± 0.002	+	–	–	25 ± 1.2	–	–	–	28 ± 1.24
**62**	MA37	49.14 ± 2.25	ND	3.83 ± 0.10	86.33 ± 0.000	+	–	–	25 ± 0.69	–	–	–	–
**63**	MA38	63.19 ± 1.19	38.35 ± 1.87	4.49 ± 0.12	65.21 ± 0.002	+	–	84.375 ± 1.02	–	–	–	–	–
**64**	MA40	62.83 ± 1.22	42.62 ± 6.7	4.45 ± 0.13	63.44 ± 0.002	+	–	90.625 ± 1.32	–	–	–	–	–
**65**	MA42	61.23 ± 1.40	6.21 ± 0.45	4.46 ± 0.14	50.07 ± 0.015	–	+	–	–	–	–	–	–
**66**	MA43	15.12 ± 0.31	4.65 ± 1.45	4.66 ± 0.12	60.22 ± 0.002	+	–	–	–	–	–	–	–
**67**	MA45	86.18 ± 1.0	8.845 ± 1.23	4.46 ± 0.11	48.99 ± 0.002	+	+	87.5 ± 1.1	–	–	–	–	–
**68**	MA48	ND	ND	0.39 ± 0.04	61.14 ± 0.001	–	–	–	–	–	–	–	30 ± 0.28
**69**	MA51	42.77 ± 1.02	ND	4.52 ± 0.10	48.59 ± 0.006	+	+	–	–	–	–	–	34 ± 0.78
**70**	MA54	58.08 ± 1.36	15.41 ± 1.12	4.49 ± 0.10	ND	–	–	–	–	–	–	–	–
**71**	MA57	ND	ND	4.40 ± 0.11	63.60 ± 0.002	–	–	–	–	–	–	–	–
**72**	MA63	8.53 ± 0.63	ND	4.81 ± 0.03	34.81 ± 0.006	+	–	–	–	–	–	–	–
**73**	MA66	24.18 ± 0.84	ND	4.69 ± 0.07	69.48 ± 0.002	–	–	–	–	–	–	–	–
**74**	MA68	81.57 ± 2.84	ND	1.08 ± 0.08	ND	–	–	–	–	–	–	–	–
**75**	MA69	52.74 ± 1.03	ND	4.65 ± 0.08	53.72 ± 0.002	–	–	–	–	–	–	–	–
**76**	MA70	21.78 ± 0.72	ND	4.46 ± 0.10	ND	–	–	–	–	–	–	–	–
**77**	MA71	6.08 ± 0.71	ND	4.70 ± 0.06	46.36 ± 0.003	–	–	–	–	–	–	–	–
**78**	K27	26.48 ± 1.22	ND	3.00 ± 0.14	ND	+	+	–	–	–	–	–	–
**79**	K33	ND	ND	0.61 ± 0.26	ND	+	–	–	–	–	–	–	–
**80**	K34	ND	ND	0.85 ± 0.26	ND	+	–	–	–	–	–	–	–
**81**	K78	16.05 ± 1.04	ND	1.48 ± 0.13	ND	+	+	–	–	–	–	–	–
**82**	K86	23.79 ± 1.60	ND	1.50 ± 0.30	67.11 ± 0.09	+	+	–	–	–	–	–	–
**83**	K91	11.14 ± 8.85	ND	4.50 ± 0.19	14.09 ± 0.52	–	+	–	–	–	–	–	–
**84**	K92	5.69 ± 1.84	ND	2.63 ± 0.20	24.43 ± 0.85	–	+	–	–	–	–	–	–
**85**	M15	14.26 ± 1.40	ND	4.92 ± 0.19	ND	–	+	–	–	–	–	–	–
**86**	M18	21.35 ± 2.17	27.36 ± 1.19	2.54 ± 0.41	ND	–	+	–	–	–	–	–	–
**87**	M34	8.10 ± 1.10	ND	4.32 ± 0.31	ND	+	+	–	–	–	–	–	–
**88**	M36	3.09 ± 1.20	ND	2.65 ± 0.18	ND	–	+	–	–	–	–	–	–

*The values represent mean value (n=3), ± (standard deviation, SE), (+) activity or growth, (-) represents negative activity or no growth. IAA - Indole Acetic Acid production; P solubilization - Phosphate solubilization; 1 - *F. oxysporum* (MTCC 284); 2 - *P. hypobrunnea* (ITCC 4141); 3 - *R. solani* (MTCC 4633); 4 - *N. sphaerica* (KJ767520); 5 - *P. lamaensis* (ITCC 292); 6 - *E. vexans* (ITCC 938). ND, not defined.

### 3.4 *In vitro* assessment of antagonistic activity

**Figure 4:**
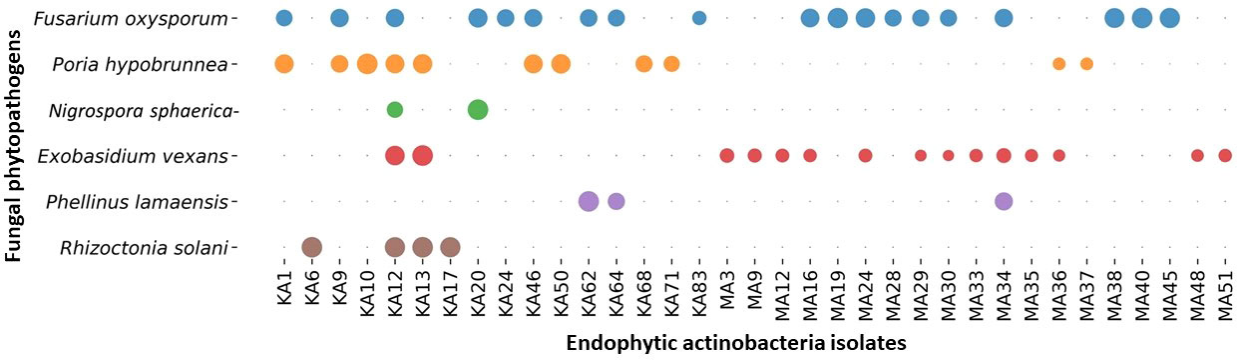
Bubble plot showing antifungal activity. The bubbles represent the antifungal activity of actinobacteria against phytopathogens. The greater the bubble size is, the greater the zone of inhibition (mm). The zone of inhibition falls in the range of 40 ± 0.82 - 10 ± 0.69.

Among the 88 EnA isolates, 35 (39.77%) of the isolates were observed to exhibit antifungal activities against at least one of the tested pathogens. The highest inhibition activity was ascertained against *Fusarium oxysporum*, followed by *Exobasidium vexans* and *Poria hypobrunnea* (**Figure 4** and **Figure S2** **).** The isolate KA12 showed extensive antifungal activity against most of the tested pathogens. Additionally, the highest reduction in mycelial growth of 90.62% was exhibited by isolate MA40 against *F. oxysporum*, followed by KA62 against *P. lamaensis* (90%), KA13 against *E. vexans* (80%), KA13 against *R. solani* (75%), KA20 against *N. sphaerica* (67.86%), and KA10 against *P. hypobrunnea* (65%) (**Table 2** **)**.

### 3.5 Chitinase enzyme production

Chitin, a polymer of 1,4-β-linked N-acetyl-D-glucosamine, is the building block of fungal cell walls. Chitinases are enzymes that degrade chitin and exhibit antagonism against fungus. Thus, actinobacteria with chitinolytic activity will be beneficial for the biocontrol of pathogens. In our study, *in vitro* screening of chitinolytic activity by the EnA isolates was determined using substrate chitin in the medium. 51 (57.95%) isolates showed chitinase enzyme production by forming clear halo zones around the colonies (**Table 3** **).**

**Table 3:** Isolates showing chitinase enzyme activity.

Sl. No.	Isolate code	Chitinase enzyme activity
**1**	KA1	+
**2**	KA3	+
**3**	KA4	+
**4**	KA5	+
**5**	KA6	+
**6**	KA9	+
**7**	KA10	+
**8**	KA11	+
**9**	KA12	+
**10**	KA13	+
**11**	KA16	+
**12**	KA17	+
**13**	KA20	+
**14**	KA24	+
**15**	KA27	+
**16**	KA28	+
**17**	KA30	+
**18**	KA31	+
**19**	KA38	+
**20**	KA40	+
**21**	KA45	+
**22**	KA46	+
**23**	KA50	+
**24**	KA51	+
**25**	KA52	+
**26**	KA61	+
**27**	KA62	+
**28**	KA64	+
**29**	KA68	+
**30**	KA70	+
**31**	KA71	+
**32**	KA76	+
**33**	KA82	+
**34**	MA3	+
**35**	MA8	+
**36**	MA9	+
**37**	MA10	+
**38**	MA11	+
**39**	MA12	+
**40**	MA13	+
**41**	MA16	+
**42**	MA19	+
**43**	MA24	+
**44**	MA30	+
**45**	MA33	+
**46**	MA34	+
**47**	MA35	+
**48**	MA37	+
**49**	MA38	+
**50**	MA42	+
**51**	MA63	+
**52**	MA66	+
**53**	MA69	+
**54**	MA70	+
**55**	MA71	+

### 3.6 Ranking of isolates based on *in vitro* PGP and biocontrol screening

In order to select the most performing EnA isolates, the isolates were organized hierarchically (a non-arbitrary ranking approach). The strains were ranked based on *in vitro* screening for their potential to promote plant growth and biocontrol ability. The obtained ranking was compared to the bonitur scale, where all the PGP and biocontrol attributes were allocated points. The utmost attainable score here is 29 points: IAA production and phosphate solubilization were each given 3 points, nitrogen fixation, ammonia, siderophore, ACC deaminase, and chitinase production were given 1 point each. Depending on the percentage of inhibition, antifungal activity against six tested pathogens was assigned 3 points each leading to a sum of 18 points (**Table 4** and **Table S3**).

**Table 4:** Bonitur ranking of top 10 endophytic actinobacteria associated with tea with PGP and biocontrol traits.

Isolate code	PGP traits					Antifungal mechanisms		Biocontrol traits						Total assessment score(29)	Rank
	IAA^a^	PS^b^	AM^c^	NF^d^	ACC^e^	Sid^f^	Chi^g^	Fo^h^	Ph^i^	Rs^j^	Ns^k^	Pl^l^	Ev^m^		
**KA12**	3	2	1	1	1	1	1	3	2	3	2	0	3	23	1^st^
**KA13**	3	0	0	1	1	1	1	0	2	3	0	0	3	15	2^nd^
**MA34**	3	2	1	1	1	1	1	3	0	0	0	0	2	15	3^rd^
**KA20**	3	0	1	1	0	1	1	3	0	0	3	0	0	13	4^th^
**KA46**	3	0	1	1	1	1	1	3	2	0	0	0	0	13	5^th^
**MA16**	0	3	1	1	1	1	1	3	0	0	0	0	1	12	6^th^
**MA30**	3	2	1	0	1	1	1	2	0	0	0	0	1	12	7^th^
**MA38**	3	2	1	1	0	1	1	3	0	0	0	0	0	12	8^th^
**KA83**	3	2	1	1	1	1	0	2	0	0	0	0	0	11	9^th^
**MA40**	3	2	1	1	0	1	0	3	0	0	0	0	0	11	10^th^

IAA^a^, Indole acetic acid production; PS^b^, Phosphate solubilization; AM^c^, Ammonia production; NF^d^, Nitrogen fixation; ACC^e^, ACC deaminase production; Sid^f^, Siderophore production; Chi^g^, Chitinase production; Fo^h^, *F. oxysporum* (MTCC 284); Ph^i^, *P. hypobrunnea* (ITCC 4141); Rs^j^, *R. solani* (MTCC 4633); Ns^k^, *N. sphaerica* (KJ767520); Pl^l^, *P. lamaensis* (ITCC 292); Ev^m^, *E. vexans* (ITCC 938).

### 3.7 Biofilm formation ability of endophytes

**Figure 5:**
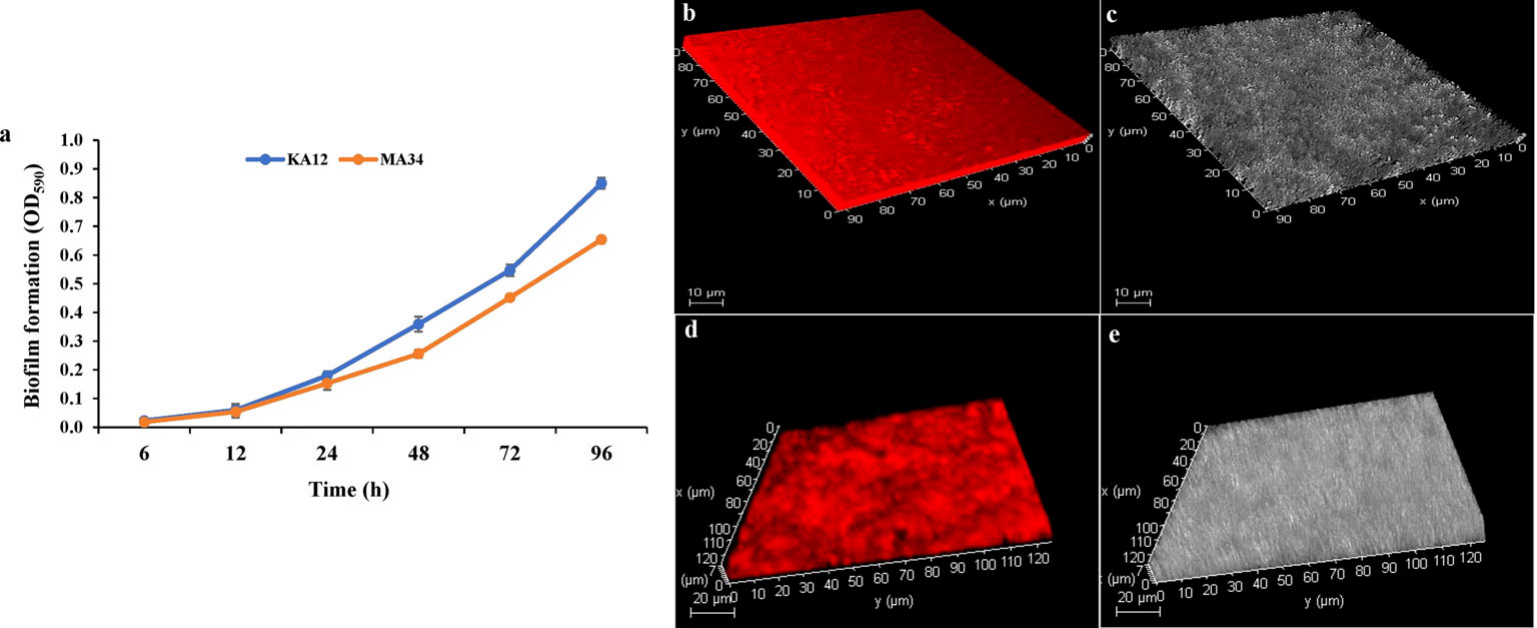
Biofilms measured with the crystal violet staining method and confocal laser scanning microscopy (CLSM). **(A)** Quantification of biofilm production at 96 h by KA12 and MA34 after elution of crystal violet stain by ethanol. Three-dimensional (3D) views of **(B, C)** KA12, **(D, E)** MA34 biofilms grown for 96 h. Actinobacteria were stained with acridine orange and observed by CLSM.

Biofilm formation by the potent EnA strains KA12 and MA34 was found to be strong during the stationary growth phase at 96 h. The significant biofilm formation, as examined by the crystal violet staining method, started after 24 h of incubation (**Figure 5A**). Along with this, CLSM studies confirmed the biofilm formation on glass slides. The biofilms formed in the glass slides by KA12 and MA34 appeared to be 9 µm and 7 µm thick, respectively, as demonstrated in the z-plane by CLSM microscopy (**Figures 5B–E**).

### 3.8 Biopriming with endophytic actinobacteria KA12 inoculant enhances the plant health

**Figure 6:**
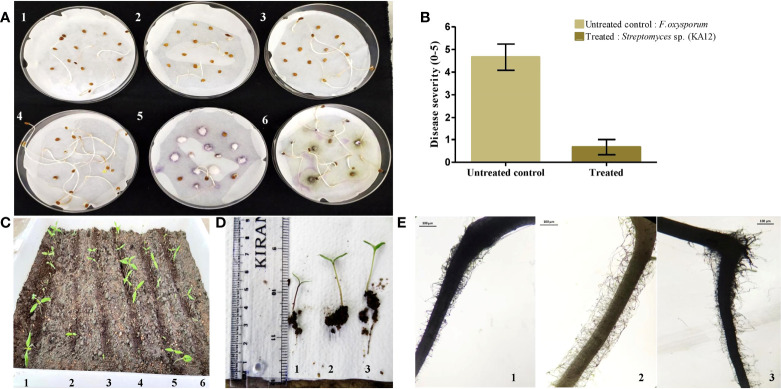
Evaluation of induced systemic resistance and seed germination. **(A)**. Seed germination plate assay (1,2,3) Control. Non-bacterized tomato seeds, (4) KA12 bacterized seeds, (5) *F*. *oxysporum* inoculated seeds, (6) KA12 bacterized seeds, and *F*. *oxysporum* inoculated **(B)** Degree of disease severity in control and inoculated seeds. **(C)** In-planta seed germination assay. (1) Bacterized tomato seeds, (2, 3, 5, 6) non-bacterized control seeds, (4) *F*. *oxysporum* inoculated seeds, **(D)** growth after 14 days of germination, (1) un-inoculated control, (2) KA12 and *F*. *oxysporum* inoculated, (3) KA12 inoculated. **(E)** Stereomicroscopic visualization of root hairs after 14 days of seed germination (1) Un-inoculated control (2) KA12 inoculated (3) Inoculated with KA12 and *F*. *oxysporum*. Scale 100μm.

Plant growth-promoting actinobacteria can also act as a defensive symbiont by biopriming response. In this study, strain *Streptomyces* sp. (KA12) was found to have potential antagonistic activity against phytopathogens viz. *F. oxysporum, P. hypobrunnea, E. vexans, R. solani*, and *N. sphaerica.* Hence, KA12 was evaluated as seed biopriming agent. The seed germination rate (%) of bacterized tomato seeds showed enhanced germination of 80 ± 0.43 compared to the non-bacterized control seeds, which were 20 ± 0.28 – 26.6 ± 0.4. The seeds inoculated with test isolate and fungal pathogen showed a seed germination rate (%) of 73.33 ± 0.25 (**Figure 6A** and **Table S4**). The severity of disease by inoculating *F. oxysporum* in bacterized tomato seeds was reduced to a scale of 0.6 compared to the non-bacterized seeds with disease severity of 4.66 (**Figure 6B**). The results obtained by conducting both pot experiments and water agar plate assay displayed resemblance confirming the biopriming effect of KA12 and effectiveness for fungal resistance (**Figures 6C, D**).

On the visualization of root architecture using a stereomicroscope, variation of root morphology was observed among treated and control groups. The number of root hairs in the control group was less than that compared to the roots colonized with KA12. The number of roots in the treatment group with KA12 and the fungal pathogen was also more than in the untreated control (**Figure 6E**). Thus, this indicates that the isolate KA12 enhances the root hair formation, thereby improving the soil anchorage and positively influencing growth.

### 3.9 Assessment of *in vivo* plant growth promotion

**Figure 7:**
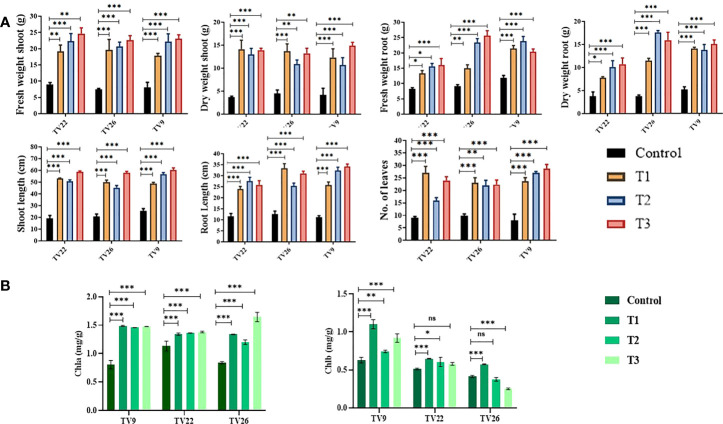
Evaluation of PGP experiment to show the effect of treatments of endophytic actinobacterial inoculum in tea clones TV22, TV26, and TV9. **(A)** vegetative parameters **(B)** chlorophyll (a, b) Error bar represents the standard error of means of replicates (*p < 0.05, **p < 0.01, ***p < 0.001). ns-not significant.

In all the experiments performed in nursery condition, on comparison of the treated plants to the control plants, a significant (p < 0.05) increase in all the vegetative parameters were observed (**Figure 7** and **Figure S3**). Further, the level of growth in vegetative parameters of treated plants correlated to control plants was determined by fold change analysis. The fold change level varied with the various EnA treatments in the tea clones used for the study. In all the tea clones TV9, TV22, and TV26, the actinobacterial inoculum of *Streptomyces* sp. KA12 (T1) showed a significant increase in growth parameters in terms of fold change compared to other inoculums. In the TV9 clone, T1 enhanced fresh and dry biomass of roots by 4.42-fold and 3.8-fold, and shoot biomass by 4.15 and 3.6-fold, respectively, root and shoot length was increased by 4.68-fold and 5.6-fold, respectively, and leaf number was increased by 4.56-fold. However, the degree of growth promotion in TV22 and TV26 clones varied with various treatments. In the TV22 clone, enhancement in shoot biomass (3.38-fold) and leaf number (4.17-fold) were observed in treatment with T1. The increase in root length (3.98-fold) was observed in treatment with T2. The enhancement in root biomass (2.79-fold) and shoot length (5.29-fold) were observed in treatment with T3. In the TV26 clone, an increase in shoot biomass (3.2-fold), root length (4.38-fold), and leaf number (3.7-fold) were observed in treatment with T1. The enhancement in root biomass (3.78-fold) was observed in treatment with T2, and the increase in shoot length (5.22-fold) was observed in treatment with T3. All these growth augmentations were observed compared to the untreated control plants. The graph of fold change analysis and their log values are provided in **Table 5** and **Figure S4**.

**Table 5:** Fold change analysis of tea clones TV9, TV22 and TV26 treated with actinobacterial strains.

Growth parameters	Log2(FC)								
	Treatment 1 (KA12)			Treatment 2 (MA34)			Treatment 3 (KA12 + MA34)		
	TV9	TV22	TV26	TV9	TV22	TV26	TV9	TV22	TV26
**Fresh weight shoot**	4.157	3.341	3.605	3.814	3.737	3.722	3.900	3.967	3.923
**Dry weight shoot**	3.624	3.388	3.202	2.693	3.222	2.671	3.415	3.360	3.127
**Fresh weight root**	4.424	2.341	2.544	3.581	2.888	3.824	3.082	2.951	4.041
**Dry weight root**	3.821	2.000	2.939	3.110	2.663	3.780	3.312	2.794	3.597
**Shoot Length**	5.603	5.073	4.871	4.960	4.970	4.623	5.115	5.298	5.222
**Root Length**	4.684	3.624	4.385	4.401	3.985	3.689	4.524	3.824	4.212
**Number of leaves**	4.565	4.170	3.700	4.248	2.807	3.585	4.369	3.907	3.624

**Figure 8:**
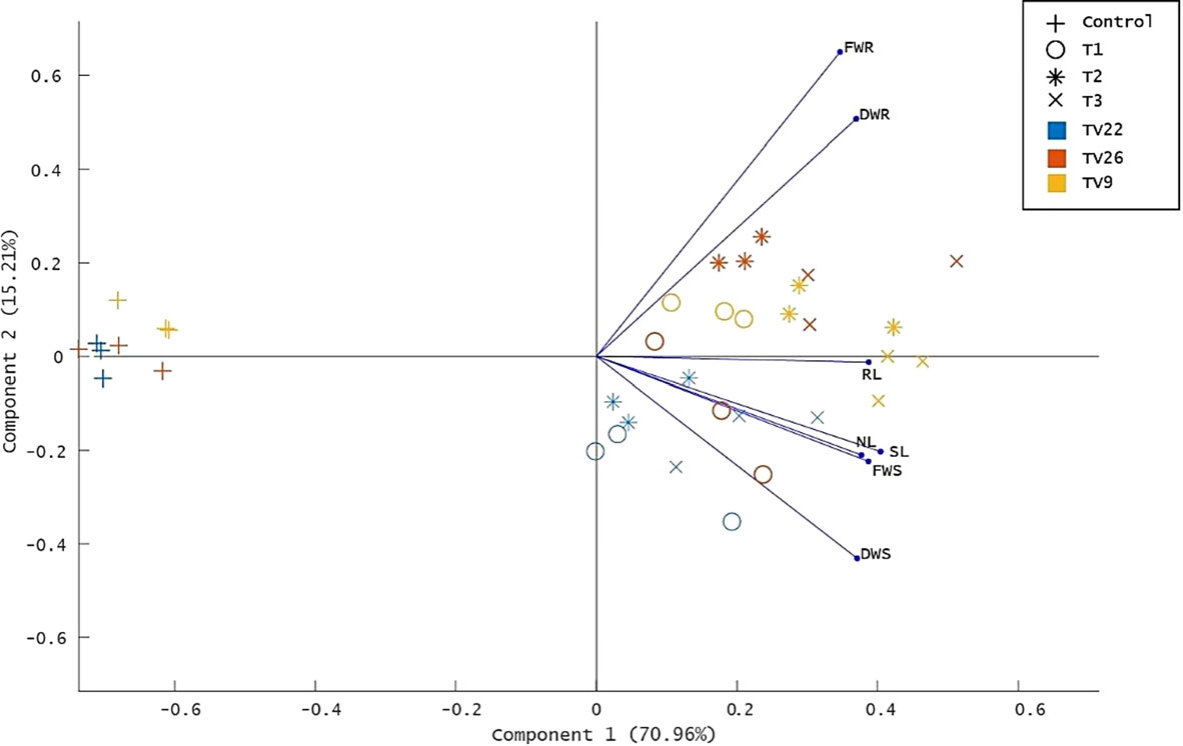
Principle component analysis biplot based on correlation matrices of the PGP dataset of growth parameters and treatment groups. Tea clones - TV9, TV22, and TV26. C- Control, Untreated plant; T1-Treatment 1, KA12; T2- Treatment 2, MA34; T3- Treatment 3, Consortia of KA12 and MA34. PCA resulted in two major principal components (PC) with a variance of 70.96% (PC1) and 15.21% (PC2) on reduction of the dimension of the various PGP parameters. FWR, fresh weight root; DWR, dry weight root; RL, root length; SL, shoot length; NL, number of leaves, FWS, fresh weight shoot; DWS, dry weight shoot.

The various PGP parameters were reduced to two principal components, PC1 and PC2, by principal component analysis (PCA) analysis. A maximum variance of 70.96% was found in PC1, followed by 15.21% in PC2 (**Figure 8** **)**. As indicated in the PCA plot, a distinct variance was observed throughout the PC1 between treated and control groups as the treatments formed separate clusters in all the tea clones used. The degree of correlation of parameters fresh and dry weight root (FWR and DWR) was represented toward clones TV9 and TV26 in all the treatment groups. Likewise, in all the treatment groups, fresh and dry weight shoot parameters, root length, the number of leaves, and shoot length were correlated mostly toward clone TV22.

### 3.10 Colonization of KA12 and MA34 in plants

The extent of effective colonization of symbiotic actinobacteria determines the influence of plant-plant growth-promoting bacteria interactions on plant growth and yield. Thus, we assessed the efficiency of *Streptomyces* sp. KA12 to colonize tomato plant by determining the CFU, which was found to be 7.2 x 10^3^ CFU/g of the root after inoculating tomato plantlets for 15 days. However, sparse actinobacterial colonies of 1.1 x 10^2^ CFU/g were recovered from control plants. On the assessment of the efficiency of *Streptomyces* sp. KA12 and *Streptomyces* sp. MA34 to colonize tea plants, CFU was found to be 4.7 x 10^3^ and 4.1 x 10^3,^ respectively.

### 3.11 Chlorophyll estimation

In the treatment group, tea clones showed an increase in chlorophyll content. Comparing the chlorophyll a and b content of the treated tea clones to the control group, the variance was found to be significant at p < 0.05 (**Table 6**; **Figure 7**). This denotes the impact of prospective actinobacteria on photosynthesis and plant development.

**Table 6:** Growth parameters of tea clones inoculated with two potent actinobacterial strains in nursery conditions.

Tea Clones	Treatment	FWS(g)	DWS(g)	FWR(g)	DWR(g)	SL(cm)	RL(cm)	NL	Chla(mg/g)	Chlb(mg/g)
**TV9**	**Control**	8.13 ± 1.503	4.20 ± 1.419	11.90 ± 0.737	5.17 ± 0.612	25.67 ± 1.856	11.17 ± 0.726	8.00 ± 2.517	0.81 ± 0.042	0.63 ± 0.023
	**T1**	17.83 ± 0.726	12.33 ± 1.881	21.47 ± 0.926	14.13 ± 0.240	48.60 ± 1.249	25.70 ± 1.457	23.67 ± 1.453	1.49 ± 0.004	1.10 ± 0.035
	**T2**	22.20 ± 2.369	10.67 ± 1.618	23.87 ± 1.489	13.80 ± 1.200	56.80 ± 1.274	32.30 ± 1.589	27.00 ± 0.577	1.46 ± 0.001	0.74 ± 0.010
	**T3**	23.07 ± 1.224	14.87 ± 0.769	20.37 ± 0.899	15.10 ± 0.850	60.33 ± 1.856	34.17 ± 1.167	28.67 ± 1.764	1.48 ± 0.001	0.92 ± 0.032
**TV22**	**Control**	9.00 ± 0.577	3.67 ± 0.240	8.27 ± 0.371	3.80 ± 0.917	19.33 ± 2.333	11.67 ± 1.202	9.00 ± 0.577	1.13 ± 0.050	0.51 ± 0.007
	**T1**	19.13 ± 1.988	14.13 ± 1.937	13.33 ± 0.882	7.80 ± 0.200	53.00 ± 0.577	24.00 ± 1.155	27.00 ± 2.646	1.34 ± 0.012	0.65 ± 0.002
	**T2**	22.33 ± 2.333	13.00 ± 1.311	15.67 ± 0.882	10.13 ± 1.338	50.67 ± 1.202	27.50 ± 1.803	16.00 ± 1.155	1.36 ± 0.003	0.61 ± 0.036
	**T3**	24.63 ± 1.770	13.93 ± 0.406	16.00 ± 2.157	10.73 ± 1.338	58.67 ± 0.882	25.83 ± 1.922	24.00 ± 1.528	1.38 ± 0.009	0.58 ± 0.011
**TV26**	**Control**	7.50 ± 0.289	4.50 ± 0.764	9.17 ± 0.441	3.83 ± 0.203	20.67 ± 2.186	12.50 ± 1.443	10.00 ± 0.577	0.84 ± 0.012	0.41 ± 0.008
	**T1**	19.67 ± 3.180	13.70 ± 1.595	15.00 ± 1.079	11.50 ± 0.500	49.93 ± 1.733	33.40 ± 2.078	23.00 ± 2.082	1.34 ± 0.004	0.58 ± 0.004
	**T2**	20.70 ± 1.300	10.87 ± 0.926	23.33 ± 1.302	17.57 ± 0.433	45.30 ± 1.767	25.40 ± 1.274	22.00 ± 2.082	1.20 ± 0.024	0.38 ± 0.014
	**T3**	22.67 ± 1.333	13.23 ± 1.102	25.63 ± 1.592	15.93 ± 1.713	58.00 ± 1.155	31.03 ± 1.017	22.33 ± 1.856	1.65 ± 0.047	0.25 ± 0.007

T1, Inoculation with *Streptomyces* sp. KA12; T2, Inoculation with *Streptomyces* sp. MA34; T3, Inoculation with consortia of KA12 and MA34; FWS, fresh weight shoot; DWS, dry weight shoot; FWR, fresh weight root; DWR, dry weight root; SL, shoot length; RL, root length; NL, number of leaves; Chla, chlorophyll a; Chlb, Chlorophyll b. Data are the means ± SE of three replicates. Mean values were significantly different at p < 0.05.

### 3.12 Effect of endophytes on element uptake

The results of elemental analysis are listed in **Table 7**. The endophytic actinobacteria inoculation affected the plant uptake of P, N, Fe, Cu, Zn, Na, Ni, Mg, Mn, and Ca. It was revealed that the inoculation with *Streptomyces* sp. KA12 and MA34 and their consortia significantly (p < 0.05) increased the uptake of these elements. The shoot and root biomass, number of leaves did not differ between treatment at harvest, but they significantly increased as compared to the untreated control tea plants, thereby influencing plant growth promotion (**Table 6**; **Figure 7**).

**Table 7:** Element concentration in the shoots of tea clones inoculated under nursery conditions with potent actinobacterial strains.

Tea Clones	Treatment	P(%)	N(%)	Fe(g/100g)	Cu(g/100g)	Zn(g/100g)	Na(g/100g)	Ni(g/100g)	Mg(g/100g)	Mn(g/100g)	Ca(g/100g)
**TV9**	**Control**	0.08 ^a^ ± 0.014	4.59 ^a^ ± 0.037	32.90 ± 0.090	1.61^a^ ± 0.006	0.82 ^a^ ± 0.023	2.71 ^a^ ± 0.083	0.16 ^a^ ± 0.001	1.40 ^a^ ± 0.005	5.19 ^a^ ± 0.005	32.74 ± 0.147
	**T1**	0.16 ^a^ ± 0.050	4.83 ± 0.039	34.90 ± 0.116	1.70 ^a^ ± 0.035	0.97 ± 0.049	3.85 ± 0.075	0.17 ^a^ ± 0.008	1.55 ^a^ ± 0.055	5.33 ^a^ ± 0.075	46.80 ± 0.579
	**T2**	0.10 ^a^ ± 0.018	4.67 ^a^ ± 0.038	34.67 ± 0.175	1.70 ^a^ ± 0.004	0.88 ^a^ ± 0.014	3.20 ^a^ ± 0.005	0.18 ^a^ ± 0.004	1.41 ^a^ ± 0.089	5.31 ^a^ ± 0.004	43.86 ± 0.093
	**T3**	0.09 ^a^ ± 0.008	4.85 ± 0.031	36.72 ± 0.670	1.84 ± 0.076	1.00 ± 0.037	3.84 ± 0.032	0.17 ^a^ ± 0.004	1.47 ^a^ ± 0.026	5.46 ^a^ ± 0.029	39.65 ± 0.334
**TV22**	**Control**	0.08 ^a^ ± 0.015	4.72 ^a^ ± 0.035	54.44 ^a^ ± 0.382	9.55 ^a^ ± 0.030	0.48 ± 0.032	2.50 ± 0.006	0.21^a^ ± 0.019	1.41^a^ ± 0.008	4.77 ^a^ ± 0.026	13.21 ± 0.006
	**T1**	0.18 ^a^ ± 0.042	4.78 ^a^ ± 0.021	59.99 ± 0.086	10.90 ± 0.122	0.89 ± 0.012	2.81 ± 0.079	0.24 ^a^ ± 0.008	1.43^a^ ± 0.004	5.19 ± 0.005	16.12 ± 0.287
	**T2**	0.14 ^a^ ± 0.004	4.79 ^a^ ± 0.034	56.98 ^a^ ± 0.225	10.79 ^a^ ± 0.345	0.82 ± 0.030	3.21 ± 0.089	0.24 ^a^ ± 0.002	1.49 ± 0.019	4.88 ^a^ ± 0.062	15.47 ± 0.042
	**T3**	0.12 ^a^ ± 0.037	4.78 ^a^ ± 0.021	57.06 ± 0.074	10.09 ^a^ ± 0.030	0.59 ± 0.011	2.89 ± 0.007	0.26 ^a^ ± 0.006	1.49 ± 0.015	5.00 ± 0.048	14.29 ± 0.007
**TV26**	**Control**	0.08 ^a^ ± 0.006	4.60 ^a^ ± 0.007	60.23 ± 0.404	7.88 ^a^ ± 0.071	0.90 ^a^ ± 0.004	2.70 ^a^ ± 0.177	0.20 ^a^ ± 0.005	1.42 ^a^ ± 0.060	4.38 ^a^ ± 0.031	10.02 ± 0.008
	**T1**	0.11 ^a^ ± 0.010	4.62 ^a^ ± 0.006	62.06 ± 0.122	8.43 ^a^ ± 0.058	1.01 ^a^ ± 0.029	2.98 ^a^ ± 0.049	0.24 ^a^ ± 0.011	1.45 ^a^ ± 0.030	4.67 ^a^ ± 0.020	13.07 ± 0.048
	**T2**	0.10 ^a^ ± 0.010	4.67 ± 0.019	61.06 ± 0.046	8.34 ^a^ ± 0.245	1.06 ± 0.030	2.87 ^a^ ± 0.074	0.21 ^a^ ± 0.005	1.49 ^a^ ± 0.015	4.55 ^a^ ± 0.124	11.62 ± 0.215
	**T3**	0.17 ± 0.021	4.64 ^a^ ± 0.013	64.77 ± 0.145	9.00 ± 0.025	0.92 ^a^ ± 0.022	2.95 ^a^ ± 0.048	0.22 ^a^ ± 0.027	1.44 ^a^ ± 0.002	4.68 ^a^ ± 0.172	11.08 ± 0.015

T1, Inoculation with *Streptomyces* sp. KA12; T2, Inoculation with *Streptomyces* sp. MA34; T3, Inoculation with consortia of KA12 and MA34;

Data are the means ± SE of three replicates. Mean values were significantly different at p<0.05. Small letter superscript with the same letter within a column and a tea clone indicates that the mean values were not significant at p < 0.05.

## 4 Discussion

The beneficial plant actinobacteria colonizing the inner parts of plants are known to improvise plant growth and development by direct or non-direct routes (Glick, 2012). They ameliorate plant nutrient uptake and provide plant defense by manifesting plant growth-promoting and antimycotic traits (Qin et al., 2011). Endophytic bacteria isolated from tea roots have been found to have a variety of PGP traits (Nath et al., 2013), as have endophytic actinobacteria isolated from wild tea species (*C. sinensis, C. japonica, C. sasanqua, C. rosiflora, C. drupifera*, and *Eurya japonica*) (Borah and Thakur, 2020); however, little is known about the endophytic actinobacterial community associated with the tea vegetative clones grown in Northeastern India.

The endophytic actinobacteria community associated with tea vegetative clones grown in Northeastern India has been studied poorly.

In this study, 88 endophytic actinobacteria were recovered from five distinct tea clones, the majority of which belonged to root tissues, which is consistent with previous studies that indicated actinobacteria dominance in root tissues (Nimnoi et al., 2010; Golinska et al., 2015; Passari et al., 2017). The presence of actinobacteria in roots is much more common than in other tissues. This emphasizes the fact that actinobacteria are natural soil dwellers that readily interact with plant roots and may form a symbiotic relationship by entering plant tissues (Sardi et al., 1992; Naik et al., 2009). Among the isolates, maximum isolates belonged to the genus *Streptomyces* (n = 69, 78.4%), which was reported in earlier studies (Cao et al., 2004). We also identified some other genera, namely *Microbacterium, Curtobacterium, Janibacter, Rhodococcus, Nocardia, Gordonia, Nocardiopsis*, and *Kribbella.* All these genera have been reported earlier to be isolated as endophytes from various plants, namely medicinal plants, apricot tree (*Pittosporum angustifolium*), *Avicennia marina, Aegiceras corniculatum, Kandelia obovota, Bruguiera gymnorrhiza, Thespesia populnea*, and *Halimione portulacoides.* (Zhao et al., 2011; Kaewkla and Franco, 2013; Jiang et al., 2018; Li et al., 2018).

In the present study, 24 (27.27%) isolates were observed to have the capacity to dissolve tri-calcium phosphate in the liquid NBRIP medium. The highest dissolved P was found in *Streptomyces* sp. MA16 and *Streptomyces* sp. KA12 with the amount of 62.19 µg/ml and 51.44 µg/ml, respectively (**Table 2**). The lowest amount of dissolved P was found in *Streptomyces* sp. KA38 with the amount of 2.48 µg/ml. These results are in agreement with the findings of (Panda et al., 2015), who reported P-solubilizing ability by bacteria in the range of 11.07 ± 0.91 - 82.77 ± 0.96 mg/l. In our study, we could also observe that most of the P-solubilizing actinobacteria belonged to the genus *Streptomyces*. Similarly, (Dastager and Damare, 2013) showed that several actinobacteria belonging to genera *Streptomyces, Kocuria, and Microbacterium* can dissolve tri-calcium phosphate and make it available to plants. Several other authors previously reported that *Streptomyces* are the most efficient phosphate solubilizers (Boubekri et al., 2021; Vargas Hoyos et al., 2021). Endophytic actinobacteria produce plant growth hormones, namely IAA, which may play an essential role in root and shoot elongation (Khamna et al., 2010). In our study, we observed 59 (67.05%) isolates as potential IAA producers in the presence of L-Tryptophan. *Streptomyces* strain KA12 produced a maximum IAA of 143 µg/ml, followed by *Streptomyces* strains KA83, MA30, and MA34, which produced 142.75 µg/ml, 135.43 µg/ml, and 122.64 µg/ml of IAA respectively (**Table 2**). Our findings are consistent with those of (Goudjal et al., 2013), who reported IAA production of 127 µg/ml by strain *Streptomyces* sp. PT2, isolated as an endophyte from the roots of *P. turgidum*, and with those of (Passari et al., 2016a), who reported endophytic *Streptomyces* strains associated with tomatoes to produce IAA in the range 7.4 to 46.3 µg/ml. Several actinobacteria were also reported to produce IAA in the presence of L-Tryptophan in the range of 13.73-142 g/ml (Khamna et al., 2008). According to *in vitro* tests, 51 (57.95%) of the isolates can grow in an N-free medium, and 71 (80.68%) of the isolates can produce ammonia in peptone water. *Microbacterium* sp. strain M15 produced the highest ammonia (4.92 µmol/ml), followed by *Streptomyces* sp. strains MA63 and MA19, which produced 4.81 µmol/ml (**Table 2**). Three *Streptomyces* sp. isolated from rice were previously reported to produce ammonia, grow in an N-free medium, and promote plant growth (Sari et al., 2014). Ammonia production promotes indirect plant growth and can act as a triggering factor by suppressing plant pathogens (Passari et al., 2016a). Siderophore production was found in 66 (75%) of all the actinobacterial isolates. *Streptomyces* sp. strain MA37 showed maximum siderophore production of 86.33% SU in the CAS liquid medium. Siderophores are ferric iron specific chelating agents with a low molecular weight produced by bacteria and fungi under iron-limiting conditions (Neilands, 1995). Several authors reported *Streptomyces* sp. and *Nocardia* sp. as siderophore producers exhibiting plant growth promotion benefits (Rungin et al., 2012; Purushotham et al., 2018). Siderophore producers in our study mainly were *Streptomyces* sp., and they were also found to have antifungal properties (**Table 2**). As previously reported, siderophore production by endophytic actinobacteria plays a role in plant pathogen biocontrol (Glick, 2012; Rungin et al., 2012). In extreme environmental stresses, EnA residing in plants that can alleviate stress by producing ACCD can decrease the production of the stress hormone ethylene and promote plant growth (Sun et al., 2006). In this study, we found that 42 (47.73%) of the isolates produced ACCD by utilizing ACC as the sole nitrogen source available in the agar medium, with the majority belonging to the genus *Streptomyces*. This is in accordance with (Shan et al., 2018), who reported that 21.73% of actinobacterial isolates associated with *Camellia sinensis* have ACCD activity. Another finding reported that found *Streptomyces* sp. GMKU 336 strain to have the highest ACCD activity (Jaemsaeng et al., 2018). Similarly, (Passari et al., 2016a) reported that *Streptomyces* sp. DBT204, DBT207, DBT219, and DBT223 had the capacity to produce ACCD. Hence, all potential isolates with multifarious PGP traits can benefit plant growth and development.

Endophytic actinobacteria are well established as significant producers of natural products with promising antifungal properties (Strobel and Daisy, 2003; Hazarika et al., 2022). This encouraged us to explore the biocontrol properties of actinobacteria. In this study, 35 (39.77%) isolates could inhibit the growth of six fungal phytopathogens, most of which belonged to the genus *Streptomyces* (n=33, 94.28%). The ability of *Streptomyces* to synthesize several bioactive metabolites as potential antifungal agents is parallel to numerous reports (Sousa and Olivares, 2016). Most of the antagonistic activity was shown against the pathogen *Fusarium oxysporum* (n=19, 54.28%). Similar antagonistic activities were earlier reported by (Passari et al., 2015). For the biocontrol of fungal disease, chitinolytic EnA plays a vital role. Besides several factors like siderophore and ammonia production, the dynamic role of chitinase enzyme production in antagonism by degradation of the fungal cell wall has been well recognized (Singh and Gaur, 2016). Out of 88 isolates, 51 (57.95%) isolates exhibited chitinolytic activity by degradation of colloidal chitin on agar plates. Chitinolytic isolates were found to have antifungal activity against the six test fungal pathogens used in this study. This could indicate that the production of chitinase enzyme by these actinobacterial isolates played a significant role in suppressing fungal growth. The findings of our study are consistent with some previous studies that reported actinobacteria with chitinolytic activity to have antifungal properties (Roberts and Selitrennikoff, 1988; Awad et al., 2014; Singh and Gaur, 2016).

The biofilm-forming ability of endophytic microbes is an essential trait for plant-microbe interaction. In our study, we selected two *Streptomyces* strains, KA12 and MA34, to be used as bioinoculant for plant growth promotion and disease suppression. On screening for biofilm traits, we found these strains to have the ability to form strong biofilms. Thus, the combination of the PGP trait and biofilm-forming ability of these strains *in vitro* may contribute to plant growth and protect the plant from pathogens by colonizing the host and non-host plants. The similar ability of bacteria (other than actinobacteria) to form biofilm *in vitro* has been evidenced in earlier works (Danhorn and Fuqua, 2007; Ansari and Ahmad, 2018); in our case, we have demonstrated biofilm formation for EnA.

For the disease suppression experiment, we chose the tomato as the test plant. We have used fungal causal pathogens of tea and vegetable crops for *in vitro* evaluation of the antagonistic activity. Therefore, we are evaluating disease prevention by using tomato seeds. Tomatoes were chosen as a test plant for this study because they germinate easily and are susceptible to infection under both *in vitro* and *in vivo* conditions. Tea seeds have a hard seed coat, making them challenging to infect in laboratory settings *in vitro* and *in vivo*. Due to the low germination rate of tea seeds under laboratory conditions, and to avoid experimental error, we decided not to include them in our fungal biocontrol experiment. The test plant gives us better efficacy in studying the beneficial properties of the isolated endophytic actinobacteria within a definite time period.

In addition to direct antifungal activity on agar plate assay, our potent strain *Streptomyces* sp. KA12 can act as a seed biopriming agent, which triggers the development of systemic resistance, thereby providing resistance to fungal disease caused by soil borne fungal pathogen *F. oxysporum*. The extent and severity of disease were also reduced to a great extent, improved seed germination, and greatly influenced root architecture, thereby promoting plant growth. Many bacteria such as *Pseudomonas* sp., *Bacillus* sp., and *Azotobacter* sp. with beneficial traits have been well documented as bioinoculant or seed bio-priming agents and observed to improve seed germination and protect the plant from biotic stress (Jaiman et al., 2020; Mitra et al., 2021). In a report, biopriming soyabean with *Streptomyces* sp. improved seed germination rate (Sahur et al., 2020). As a result, seed biopriming can help transfer beneficial PGP actinobacteria from the lab to the soil, which improves plant growth and disease resistance.

In our current study, *in vivo* experiments on tea clones using *Streptomyces* sp. strains KA12 and MA34 revealed a significant increase in all the growth parameters evaluated. These strains are known to produce PGP traits as studied during *in vitro* assays and may have a beneficial role in the *in vivo* experiment. The treatments showed enhanced biomass, height, and leaf number compared to the control. These findings are similar to earlier reports that demonstrated that actinobacteria confer benefits to host plants by boosting health and overall growth (Passari et al., 2015; Passari et al., 2016a; Vurukonda et al., 2018; Xu et al., 2021). The efficiency of the selected endophytes as PGP agents was further validated by two-way ANOVA and fold change analysis.

The effect of inoculation with the endophytes *Streptomyces* sp. (KA12) and *Streptomyces* sp. (MA34) in the nursery experiment improved the vegetative parameters of tea clones by increasing the content of chlorophylls while also increasing the content of the minerals P, N, Fe, Cu, Zn, Na, Ni, Mg, Mn, and Ca and the overall uptake by the tea clones as compared to the untreated control tea plants. All these minerals are essential nutrients for plants which influence plant growth. In our study, we demonstrated that the inoculation with strain KA12 is more efficient. These findings are consistent with previous study of (Burges et al., 2016), who demonstrated that inoculating *F. rubra* plants with endophyte *Pseudomonas* sp. had a beneficial effect. It increased several soil microbial parameters after inoculation, that also increased the chlorophyll content and aided in phytostabilization. Another study by (García-Latorre et al., 2021) described the role of fungal endophyte *S. intermedia* in nutrient uptake of Ca, Cu, Mn, Pb, Tl, and Zn in subclover. This endophyte increased the nutrient uptake thereby enhancing the nutritional value of forage. Some other studies also reported that inoculation of chickpea with phosphate solubilizing *Mesorhizobium* strains enhanced the plant P and N content (Imen et al., 2015).

Among these isolates, *Streptomyces* sp. KA12 exhibited the most significant positive effects on growth parameters and was the most effective biocontrol agent, indicating its potential use as a biofertilizer in sustainable agriculture.

## 5 Concluding remarks

In this study, the culturable endophytic actinobacteria isolated from tea vegetative clones revealed *in vitro* PGP traits, antifungal activity, and biofilm-forming capability. Since any greenhouse or field studies must be preceded by *in vitro* studies, these results provide strong evidence that endophytic actinobacteria have the potential to provide sustainable agricultural options. Thus, with the identification and characterization of these strains, we conclude that exploring endophytic actinobacteria helps us select potential strains. These strains would be a great addition to an integrated management strategy that could use eco-friendly actinobacteria to minimize the use of chemical inputs in agriculture. However, to carry out in-depth research on microbial resources associated with tea in the future, the production and synthesis of endophytes-mediated secondary metabolites, followed by multilocational trials and insights into the processes of interaction between endophytic actinobacteria and tea plants, must be assessed for their ultimate use in developing commercial bioformulation.

## Data availability statement

The datasets presented in this study can be found in online repositories. The names of the repository/repositories and accession number(s) can be found in the article/**Supplementary Material**.

## Author contributions

SH planned, designed, conducted the laboratory and field experiments, acquired, analyzed the data, interpreted the results, written and reviewed the original draft. KS contributed to data analysis. DT conceptualized and supervised the research work, reviewed and edited the original draft. All authors have read and approved the final manuscript.

## Funding

This work was partially supported by the Department of Biotechnology (DBT), Govt. of India project (Grant No.BT/PR24618/NER/95/779/2017).

## Acknowledgments

We thank the Director, Institute of Advanced Study in Science and Technology (IASST), Guwahati, Assam, India, for providing facilities for this work; Kopati Tea estate, Assam, India, Meg Tea estate, Umsning, Meghalaya, and Bateli Tea estate, Udalguri, Assam for approving and assisting in sample collection; Bioinformatics Facility (BIF), IASST for providing facilities and support for data analysis; and Sophisticated Analytical Instrument Centre (SAIC), IASST for providing infrastructure facilities to carry out part of the work.

## Conflict of interest

The authors declare that the research was conducted in the absence of any commercial or financial relationships that could be construed as a potential conflict of interest.

## Publisher’s note

All claims expressed in this article are solely those of the authors and do not necessarily represent those of their affiliated organizations, or those of the publisher, the editors and the reviewers. Any product that may be evaluated in this article, or claim that may be made by its manufacturer, is not guaranteed or endorsed by the publisher.
