# Supplementary material for: Characterization and selection of endophytic actinobacteria for growth and disease management of Tea (Camellia sinensis L.)
Source: Front Plant Sci. 2022 Nov 9;13:989794. doi: 10.3389/fpls.2022.989794 (PMC9681920; doi:10.3389/fpls.2022.989794)
Supplement: Supplementary file 1 [file Table_1.docx]

**TABLE S1.** Phenotypic morphology of endophytic actinobacteria

| Sl. No. | Isolate code | Aerial mycelia | Substrate mycelia | Pigment | Texture | Color Series |
| --- | --- | --- | --- | --- | --- | --- |
| 1 | KA1 | White | Yellow | ND | Dry | White |
| 2 | KA3 | Colorless | Colorless | ND | Patchy | White |
| 3 | KA4 | White | Off White | ND | Dry | White |
| 4 | KA5 | White | Off White | ND | Dry | White |
| 5 | KA6 | Yellowish Green | Greenish | Yellow | Dry, Patchy | Yellow |
| 6 | KA9 | Gray | Gray | ND | Dry, Patchy | Gray |
| 7 | KA10 | Gray | White | ND | Dry | Gray |
| 8 | KA11 | Gray | Off White | ND | Dry | Gray |
| 9 | KA12 | Light Yellow | Off White | Yellow | Dry, Patchy | Yellow |
| 10 | KA13 | Grayish Brown | Gray | ND | Dry, Patchy | Gray |
| 11 | KA16 | White | Yellow | ND | Patchy | White |
| 12 | KA17 | White | Off White | ND | Patchy | White |
| 13 | KA20 | Colorless | Colorless | ND | Patchy | White |
| 14 | KA24 | White | Yellow | Yellow | Dry, Patchy | White |
| 15 | KA25 | Black | Black | ND | Dry | Gray |
| 16 | KA28 | Off White | Light Brown | Brown | Patchy, Powdery | Gray |
| 17 | KA30 | Light Pink | Yellow | Light Pink | Patchy | Yellow |
| 18 | KA32 | Off White | Off White | ND | Patchy | White |
| 19 | KA33 | White | Off White | ND | Dry | White |
| 20 | KA35 | Light Brown | Light Brown | Brown | Dry, Patchy | Gray |
| 21 | KA38 | Gray | Light Brown | Light Brown | Dry | Gray |
| 22 | KA40 | Gray | Light Brown | Yellow | Patchy | Gray |
| 23 | KA41 | Peach | Peach | ND | Patchy | Red |
| 24 | KA45 | White | Light Brown | Light Brown | Dry | Gray |
| 25 | KA46 | Gray | Dark Gray | Light Brown | Dry | Gray |
| 26 | KA47 | Dark Green | Dark Green | Brown | Patchy | Yellow |
| 27 | KA50 | White | Off White | ND | Patchy, Powdery | White |
| 28 | KA51 | Light Brown | Light Brown | Brown | Dry, Patchy | Gray |
| 29 | KA52 | Off White | Light Brown | Brown | Patchy | Gray |
| 30 | KA54 | Light Brown | Light Brown | Brown | Dry | Gray |
| 31 | KA55 | White | Off White | Light Brown | Dry | White |
| 32 | KA61 | Gray | Black | ND | Dry | Gray |
| 33 | KA62 | Off White | Brown | ND | Dry | Gray |
| 34 | KA64 | Off White | Off White | ND | Patchy | White |
| 35 | KA68 | White | Off White | Black | Dry | White |
| 36 | KA71 | Gray | Black | ND | Dry | Gray |
| 37 | KA76 | White | Black | ND | Patchy | Gray |
| 38 | KA82 | Off White | Brown | Yellow | Patchy | Gray |
| 39 | KA83 | Light Yellow | Off White | Yellow | Dry, Patchy | Yellow |
| 40 | KA85 | Black | Black | ND | Dry | Gray |
| 41 | KA87 | Off White | Light Brown | Brown | Patchy, Powdery | Gray |
| 42 | KA88 | Light Pink | Yellow | Light Pink | Patchy | Yellow |
| 43 | KA89 | Gray | Black | ND | Dry | Gray |
| 44 | MA3 | Off White | Off White | ND | Patchy | White |
| 45 | MA8 | White | Light Brown | Light Yellow | Dry, Patchy | White |
| 46 | MA9 | Maroon | Maroon | Reddish | Dry, Powdery | Red |
| 47 | MA10 | Off White | Light Brown | Light Brown | Patchy | Gray |
| 48 | MA11 | Off White | Off White | Light Brown | Patchy | White |
| 49 | MA12 | Maroon | Maroon | Reddish | Patchy | Red |
| 50 | MA13 | Light Yellow | Off White | ND | Patchy | Yellow |
| 51 | MA16 | Off White | Off White | Yellow | Dry, Smooth | White |
| 52 | MA19 | Off White | Off White | Pinkish | Patchy | White |
| 53 | MA24 | Off White | Off White | Black | Dry, Smooth | White |
| 54 | MA26 | White | Off White | ND | Patchy | White |
| 55 | MA28 | White | White | ND | Patchy | White |
| 56 | MA29 | White | White | ND | Patchy | White |
| 57 | MA30 | White | White | ND | Dry | White |
| 58 | MA33 | Gray | Gray | Black | Dry | Gray |
| 59 | MA34 | Gray | Light Brown | Brown | Dry | Gray |
| 60 | MA35 | Gray | Off White | ND | Dry | Gray |
| 61 | MA36 | Gray | Gray | Brown | Dry | Gray |
| 62 | MA37 | Gray | Gray | ND | Dry | Gray |
| 63 | MA38 | Gray | Black | Black | Dry | Gray |
| 64 | MA40 | Off White | Off White | ND | Patchy | White |
| 65 | MA42 | Brown | Brown | ND | Patchy | Gray |
| 66 | MA43 | Off White | Off White | ND | Dry | White |
| 67 | MA45 | Brown | Brown | ND | Patchy | Gray |
| 68 | MA48 | Dark Brown | Dark Brown | ND | Patchy | Gray |
| 69 | MA51 | Gray | Black | ND | Patchy | Gray |
| 70 | MA54 | Light Brown | Greenish | ND | Patchy | Gray |
| 71 | MA57 | White | Off White | ND | Dry, Powdery | White |
| 72 | MA63 | Off White | Off White | ND | Dry | White |
| 73 | MA66 | Off White | Off White | ND | Patchy | White |
| 74 | MA68 | Light Brown | Light Brown | ND | Dry | Gray |
| 75 | MA69 | Peach | Off White | ND | Dry | Red |
| 76 | MA70 | White | Off White | ND | Dry, Smooth | White |
| 77 | MA71 | White | Off White | ND | Dry | White |
| 78 | K27 | Off White | Off White | ND | Dry | White |
| 79 | K33 | White | Off White | ND | Dry | White |
| 80 | K34 | Off White | Off White | ND | Dry | White |
| 81 | K78 | Peach | Off white | ND | Dry | Red |
| 82 | K86 | Light Yellow | Light Yellow | ND | Dry | Yellow |
| 83 | K91 | White | White | ND | Dry | White |
| 84 | K92 | White | Off White | ND | Dry | White |
| 85 | M15 | Yellow | Yellow | ND | Dry | Yellow |
| 86 | M18 | Light Yellow | Light Yellow | ND | Dry | Yellow |
| 87 | M34 | Peach | Off White | ND | Dry | Red |
| 88 | M36 | Off White | Off White | ND | Dry | White |

ND- not defined
