# Supplementary material for: Characterization and selection of endophytic actinobacteria for growth and disease management of Tea (Camellia sinensis L.)
Source: Front Plant Sci. 2022 Nov 9;13:989794. doi: 10.3389/fpls.2022.989794 (PMC9681920; doi:10.3389/fpls.2022.989794)
Supplement: Supplementary file 2 [file Table_2.docx]

**Table S2.** Endophytic actinobacteria isolated from Tea clones and their identification based on 16S rRNA gene sequences

| Sl. No. | Isolate code | Tea Clone | Plant Tissue | Organism Name | Type strain | Similarity (%) | GenBank, NCBI Accession No. |
| --- | --- | --- | --- | --- | --- | --- | --- |
| 1 | KA1 | TV25 | Root | *Streptomyces* sp. | *Streptomyces cyaneofuscatus* strain CSSP436 | 99.72 | OK483055.1 |
| 2 | KA3 | TV25 | Root | *Streptomyces* sp. | *Streptomyces badius* strain NBRC 12745 | 99.86 | OK483056.1 |
| 3 | KA4 | TN17 | Root | *Streptomyces* sp. | *Streptomyces pratensis* strain ch24 | 99.57 | OK483057.1 |
| 4 | KA5 | TN17 | Root | *Streptomyces* sp. | *Streptomyces badius* strain CSSP536 | 99.5 | OK483058.1 |
| 5 | KA6 | TN17 | Root | *Streptomyces* sp. | *Streptomyces pratensis* strain ch24 | 99.79 | OK483059.1 |
| 6 | KA9 | TN17 | Root | *Streptomyces* sp. | *Streptomyces pratensis* strain ch24 | 99.79 | OK483060.1 |
| 7 | KA10 | TN17 | Root | *Streptomyces* sp. | *Streptomyces pratensis* strain ch24 | 99.79 | OK483061.1 |
| 8 | KA11 | TN17 | Root | *Streptomyces* sp. | *Streptomyces pratensis* strain ch24 | 99.86 | OK483062.1 |
| 9 | KA12 | TV25 | Root | *Streptomyces* sp. | *Streptomyces pratensis* strain ch24 | 99.62 | MN483270.1 |
| 10 | KA13 | TV25 | Root | *Streptomyces* sp. | *Streptomyces pratensis* strain ch24 | 99.62 | MN483271.1 |
| 11 | KA16 | TN17 | Root | *Streptomyces* sp. | *Streptomyces anulatus* strain NBRC 13369 | 99.58 | OK483063.1 |
| 12 | KA17 | TN17 | Root | *Rhodococcus* sp. | *Rhodococcus degradans* strain CCM 4446 | 99.86 | OK483064.1 |
| 13 | KA20 | TN17 | Root | *Streptomyces* sp. | *Streptomyces anulatus* strain NBRC 13369 | 99.93 | OK483065.1 |
| 14 | KA24 | TN17 | Leaf | *Streptomyces* sp. | *Streptomyces praecox* strain NBRC 13073 | 99.86 | OK483066.1 |
| 15 | KA25 | TV25 | Leaf | *Streptomyces* sp. | *Streptomyces acrimycini* strain CSSP430 | 99.47 | MN483272.1 |
| 16 | KA28 | TN17 | Leaf | *Streptomyces* sp. | *Streptomyces caviscabies* strain ATCC 51928 | 99.77 | MN483273.1 |
| 17 | KA30 | TV22 | Leaf | *Streptomyces* sp. | *Streptomyces baarnensis* strain NBRC 14727 | 99.91 | MN493977.1 |
| 18 | KA32 | TV22 | Root | *Gordonia* sp. | *Gordonia sputi* strain 3884 | 99.71 | OK483067.1 |
| 19 | KA33 | TV22 | Root | *Kribbella* sp. | *Kribbella podocarpi* strain YPL1 | 98.94 | OK483068.1 |
| 20 | KA35 | TV22 | Root | *Streptomyces* sp. | *Streptomyces cyslabdanicus* stain K04-0144 | 98.37 | OK483069.1 |
| 21 | KA38 | TN17 | Root | *Streptomyces* sp. | *Streptomyces anulatus* strain NBRC 13369 | 99.86 | OK483070.1 |
| 22 | KA40 | TN17 | Root | *Streptomyces* sp. | *Streptomyces praecox* strain NBRC 13073 | 99.79 | OK483071.1 |
| 23 | KA41 | TN17 | Root | *Kribbella* sp. | *Kribbella podocarpi* strain YPL1 | 99.08 | OK483072.1 |
| 24 | KA45 | TV25 | Root | *Streptomyces* sp. | *Streptomyces pratensis* strain ch24 | 99.64 | OK483073.1 |
| 25 | KA46 | TV25 | Root | *Streptomyces* sp. | *Streptomyces cyslabdanicus* strain K04-0144 | 98.79 | OK483074.1 |
| 26 | KA47 | TV25 | Root | *Streptomyces* sp. | *Streptomyces olivaceoviridis* strain NBRC 13066 | 98.65 | OK483075.1 |
| 27 | KA50 | TN17 | Leaf | *Streptomyces* sp. | *Streptomyces praecox* strain CSSP720 | 99.57 | OK483076.1 |
| 28 | KA51 | TN17 | Leaf | *Streptomyces* sp. | *Streptomyces anulatus* strain NBRC 12755 | 99.43 | OK483077.1 |
| 29 | KA52 | TN17 | Leaf | *Streptomyces* sp. | *Streptomyces pratensis* strain ch24 | 99.5 | OK483078.1 |
| 30 | KA54 | TV9 | Root | *Streptomyces* sp. | *Streptomyces intermedius* strain NBRC 13049 | 99.07 | OK483079.1 |
| 31 | KA55 | TV1 | Root | *Streptomyces* sp. | *Streptomyces intermedius* strain NBRC 13049 | 98.79 | OK483080.1 |
| 32 | KA61 | TV1 | Root | *Streptomyces* sp. | *Streptomyces flavofuscus* strain NBRC 100768 | 99.84 | MN493978.1 |
| 33 | KA62 | TV1 | Root | *Streptomyces* sp. | *Streptomyces praecox* strain NBRC 13073 | 99.57 | OK483081.1 |
| 34 | KA64 | TV1 | Root | *Streptomyces* sp. | *Streptomyces praecox* strain CSSP720 | 99.29 | OK483082.1 |
| 35 | KA68 | TV9 | Root | *Streptomyces* sp. | *Streptomyces corchorusii* strain NBRC 13032 | 98.58 | OK483083.1 |
| 36 | KA71 | TV1 | Leaf | *Streptomyces* sp. | *Streptomyces pratensis* strain ch24 | 99.43 | OK483085.1 |
| 37 | KA76 | TV9 | Leaf | *Streptomyces* sp. | *Streptomyces californicus* strain CSSP711 | 99.72 | OK483086.1 |
| 38 | KA82 | TV9 | Leaf | *Streptomyces* sp. | *Streptomyces pratensis* strain ch24 | 99.51 | OK483087.1 |
| 39 | KA83 | TV25 | Root | *Streptomyces* sp. | *Streptomyces pratensis* strain ch24 | 99.72 | OK483088.1 |
| 40 | KA85 | TN17 | Leaf | *Streptomyces* sp. | *Streptomyces praecox* strain NBRC 13073 | 99.86 | OK483090.1 |
| 41 | KA87 | TN17 | Leaf | *Streptomyces* sp. | *Streptomyces anulatus* strain 13369 | 99.08 | OK483091.1 |
| 42 | KA88 | TV22 | Leaf | *Streptomyces* sp. | *Streptomyces anulatus* strain 13369 | 99.36 | OK483092.1 |
| 43 | KA89 | TV1 | Root | *Streptomyces* sp. | *Streptomyces praecox* strain NBRC 13073 | 99.57 | OK483093.1 |
| 44 | MA3 | TV9 | Leaf | *Nocardiopsis* sp. | *Nocardiopsis flavescens* strain SA6 | 99.57 | OK483116.1 |
| 45 | MA8 | TV1 | Leaf | *Streptomyces* sp. | *Streptomyces rhizosphaerihabitans* strain JR-35 | 98.94 | OK483096.1 |
| 46 | MA9 | TV1 | Leaf | *Streptomyces* sp. | *Streptomyces californicus* strain CSSP711 | 98.66 | OK483097.1 |
| 47 | MA10 | TV1 | Leaf | *Rhodococcus* sp. | *Rhodococcus degradans* strain CCM 4446 | 99.5 | OK483098.1 |
| 48 | MA11 | TV1 | Leaf | *Streptomyces* sp. | *Streptomyces rhizosphaerihabitans* strain JR-35 | 99.29 | OK483099.1 |
| 49 | MA12 | TV1 | Leaf | *Streptomyces* sp. | *Streptomyces californicus* strain CSSP711 | 99.72 | OK483100.1 |
| 50 | MA13 | TV1 | Leaf | *Streptomyces* sp. | *Streptomyces praecox* strain CSSP720 | 99.36 | OK483101.1 |
| 51 | MA16 | TV1 | Leaf | *Streptomyces* sp. | *Streptomyces rhizosphaerihabitans* strain JR-35 | 98.58 | OK483102.1 |
| 52 | MA19 | TV1 | Leaf | *Streptomyces* sp. | *Streptomyces rhizosphaerihabitans* strain JR-35 | 99.15 | OK483104.1 |
| 53 | MA24 | TV9 | Root | *Streptomyces* sp. | *Streptomyces olivochromogenes* strain NBRC 3178 | 99.08 | OK483105.1 |
| 54 | MA26 | TV9 | Root | *Streptomyces* sp. | *Streptomyces pratensis* strain ch24 | 92.1 | OK483106.1 |
| 55 | MA28 | TV9 | Root | *Streptomyces* sp. | *Streptomyces olivochromogenes* strain NBRC 3178 | 99.22 | OK483107.1 |
| 56 | MA29 | TV9 | Root | *Streptomyces* sp. | *Streptomyces olivochromogenes* strain DSM 40451 | 99.86 | OK483108.1 |
| 57 | MA30 | TV9 | Root | *Streptomyces* sp. | *Streptomyces olivochromogenes* strain NBRC 3178 | 99.64 | OK483109.1 |
| 58 | MA33 | TV1 | Root | *Streptomyces* sp. | *Streptomyces olivochromogenes* strain NBRC 13067 | 99.65 | OK483111.1 |
| 59 | MA34 | TV1 | Root | *Streptomyces* sp. | *Streptomyces mirabilis* strain CSSP107 | 99.93 | OK483112.1 |
| 60 | MA35 | TV1 | Root | *Streptomyces* sp. | *Streptomyces olivochromogenes* strain NBRC 3178 | 99.79 | OK483113.1 |
| 61 | MA36 | TV1 | Root | *Streptomyces* sp. | *Streptomyces olivochromogenes* strain DSM 40451 | 99.93 | OK483114.1 |
| 62 | MA37 | TV1 | Root | *Streptomyces* sp. | *Streptomyces mirabilis* strain NBRC 13450 | 99.29 | OK483115.1 |
| 63 | MA38 | TV22 | Root | *Streptomyces* sp. | *Streptomyces mirabilis* strain CSSP107 | 99.86 | OK483116.1 |
| 64 | MA40 | TV22 | Root | *Gordonia* sp. | *Gordonia caeni* strain MJ32 | 99.86 | OK483117.1 |
| 65 | MA42 | TV22 | Root | *Streptomyces* sp. | *Streptomyces mirabilis* strain NBRC 13450 | 99.71 | OK483118.1 |
| 66 | MA43 | TV22 | Root | *Streptomyces* sp. | *Streptomyces mirabilis* strain NBRC 13450 | 99.72 | OK483119.1 |
| 67 | MA45 | TV22 | Root | *Streptomyces* sp. | *Streptomyces olivochromogenes* strain NBRC 3178 | 99.72 | OK483120.1 |
| 68 | MA48 | TV22 | Root | *Nocardia* sp. | *Nocardia jiangxiensis* strain 43401 | 99.07 | OK483121.1 |
| 69 | MA51 | TV22 | Root | *Streptomyces* sp. | *Streptomyces olivochromogenes* strain NBRC 3178 | 99.79 | OK483122.1 |
| 70 | MA54 | TV22 | Root | *Streptomyces* sp. | *Streptomyces olivochromogenes* strain DSM 40451 | 99.71 | OK483123.1 |
| 71 | MA57 | TV22 | Root | *Nocardia* sp. | *Nocardia jiangxiensis* strain 43401 | 98.92 | OK483125.1 |
| 72 | MA63 | TN17 | Root | *Streptomyces* sp. | *Streptomyces pratensis* strain ch24 | 99.65 | OK483127.1 |
| 73 | MA66 | TN17 | Root | *Streptomyces* sp. | *Streptomyces kunmingensis* strain NBRC 14463 | 99.01 | OK483128.1 |
| 74 | MA68 | TN17 | Root | *Nocardia* sp. | *Nocardia jiangxiensis* strain 43401 | 99.07 | OK483129.1 |
| 75 | MA69 | TN17 | Root | *Streptomyces* sp. | *Streptomyces pratensis* strain ch24 | 99.65 | OK483130.1 |
| 76 | MA70 | TN17 | Root | *Streptomyces* sp. | *Streptomyces anulatus* strain 13369 | 99.65 | OK483131.1 |
| 77 | MA71 | TN17 | Root | *Streptomyces* sp. | *Streptomyces anulatus* strain 13369 | 99.79 | OK483132.1 |
