# Supplementary material for: Characterization and selection of endophytic actinobacteria for growth and disease management of Tea (Camellia sinensis L.)
Source: Front Plant Sci. 2022 Nov 9;13:989794. doi: 10.3389/fpls.2022.989794 (PMC9681920; doi:10.3389/fpls.2022.989794)
Supplement: Supplementary file 3 [file Table_3.docx]

**TABLE S3.** Bonitur ranking of endophytic actinobacteria associated with tea with PGP and biocontrol traits.

| Isolate code | PGP traits | | | | | Antifungal mechanisms | | Biocontrol traits | | | | | | Total assessment score  (29) | Rank |
| --- | --- | --- | --- | --- | --- | --- | --- | --- | --- | --- | --- | --- | --- | --- | --- |
|  | **IAA^a^** | **PS^b^** | **AM^c^** | **NF^d^** | **ACC^e^** | **Sid^f^** | **Chi^g^** | **Fo^h^** | **Ph^i^** | **Rs^j^** | **Ns^k^** | **Pl^l^** | **Ev^m^** |  |  |
| KA12 | 3 | 2 | 1 | 1 | 1 | 1 | 1 | 3 | 2 | 3 | 2 | 0 | 3 | 23 | 1^st^ |
| KA13 | 3 | 0 | 0 | 1 | 1 | 1 | 1 | 0 | 2 | 3 | 0 | 0 | 3 | 15 | 2^nd^ |
| MA34 | 3 | 2 | 1 | 1 | 1 | 1 | 1 | 3 | 0 | 0 | 0 | 0 | 2 | 15 | 3^rd^ |
| KA20 | 3 | 0 | 1 | 1 | 0 | 1 | 1 | 3 | 0 | 0 | 3 | 0 | 0 | 13 | 4^th^ |
| KA46 | 3 | 0 | 1 | 1 | 1 | 1 | 1 | 3 | 2 | 0 | 0 | 0 | 0 | 13 | 5^th^ |
| MA16 | 0 | 3 | 1 | 1 | 1 | 1 | 1 | 3 | 0 | 0 | 0 | 0 | 1 | 12 | 6^th^ |
| MA30 | 3 | 2 | 1 | 0 | 1 | 1 | 1 | 2 | 0 | 0 | 0 | 0 | 1 | 12 | 7^th^ |
| MA38 | 3 | 2 | 1 | 1 | 0 | 1 | 1 | 3 | 0 | 0 | 0 | 0 | 0 | 12 | 8^th^ |
| KA83 | 3 | 2 | 1 | 1 | 1 | 1 | 0 | 2 | 0 | 0 | 0 | 0 | 0 | 11 | 9^th^ |
| MA40 | 3 | 2 | 1 | 1 | 0 | 1 | 0 | 3 | 0 | 0 | 0 | 0 | 0 | 11 | 10^th^ |
| MA45 | 3 | 1 | 1 | 1 | 1 | 1 | 0 | 3 | 0 | 0 | 0 | 0 | 0 | 11 | 11^th^ |
| KA9 | 1 | 0 | 1 | 1 | 1 | 1 | 1 | 3 | 2 | 0 | 0 | 0 | 0 | 11 | 12^th^ |
| MA29 | 3 | 0 | 1 | 1 | 1 | 1 | 0 | 2 | 0 | 0 | 0 | 0 | 1 | 10 | 13^th^ |
| MA36 | 3 | 2 | 1 | 1 | 0 | 1 | 0 | 0 | 1 | 0 | 0 | 0 | 1 | 10 | 14^th^ |
| KA1 | 1 | 0 | 1 | 1 | 1 | 1 | 1 | 2 | 2 | 0 | 0 | 0 | 0 | 10 | 15^th^ |
| KA17 | 3 | 0 | 1 | 0 | 1 | 1 | 1 | 0 | 0 | 3 | 0 | 0 | 0 | 10 | 16^th^ |
| MA3 | 2 | 1 | 1 | 1 | 1 | 1 | 1 | 0 | 0 | 0 | 0 | 0 | 2 | 10 | 17^th^ |
| MA24 | 1 | 0 | 1 | 1 | 1 | 1 | 1 | 3 | 0 | 0 | 0 | 0 | 1 | 10 | 18^th^ |
| KA62 | 0 | 0 | 0 | 1 | 0 | 1 | 1 | 3 | 0 | 0 | 0 | 3 | 0 | 9 | 19^th^ |
| MA19 | 1 | 0 | 1 | 1 | 1 | 1 | 1 | 3 | 0 | 0 | 0 | 0 | 0 | 9 | 20^th^ |
| KA10 | 1 | 0 | 1 | 1 | 1 | 1 | 1 | 0 | 2 | 0 | 0 | 0 | 0 | 8 | 21^st^ |
| KA64 | 0 | 0 | 0 | 1 | 1 | 1 | 1 | 2 | 0 | 0 | 0 | 2 | 0 | 8 | 22^nd^ |
| MA33 | 3 | 0 | 1 | 1 | 0 | 1 | 1 | 0 | 0 | 0 | 0 | 0 | 1 | 8 | 23^rd^ |
| MA42 | 3 | 1 | 1 | 0 | 1 | 1 | 1 | 0 | 0 | 0 | 0 | 0 | 0 | 8 | 24^th^ |
| KA4 | 3 | 1 | 1 | 0 | 0 | 1 | 1 | 0 | 0 | 0 | 0 | 0 | 0 | 7 | 25^th^ |
| MA12 | 1 | 0 | 1 | 1 | 1 | 1 | 1 | 0 | 0 | 0 | 0 | 0 | 1 | 7 | 26^th^ |
| MA35 | 2 | 0 | 1 | 1 | 0 | 1 | 1 | 0 | 0 | 0 | 0 | 0 | 1 | 7 | 27^th^ |
| KA11 | 2 | 0 | 1 | 1 | 1 | 1 | 0 | 0 | 0 | 0 | 0 | 0 | 0 | 6 | 28^th^ |
| KA33 | 3 | 0 | 1 | 1 | 0 | 1 | 0 | 0 | 0 | 0 | 0 | 0 | 0 | 6 | 29^th^ |
| KA35 | 3 | 0 | 1 | 1 | 0 | 1 | 0 | 0 | 0 | 0 | 0 | 0 | 0 | 6 | 30^th^ |
| KA41 | 3 | 0 | 1 | 1 | 0 | 1 | 0 | 0 | 0 | 0 | 0 | 0 | 0 | 6 | 31^st^ |
| MA28 | 0 | 0 | 1 | 1 | 1 | 1 | 0 | 2 | 0 | 0 | 0 | 0 | 0 | 6 | 32^nd^ |
| MA51 | 1 | 0 | 1 | 1 | 1 | 1 | 0 | 0 | 0 | 0 | 0 | 0 | 1 | 6 | 33^rd^ |
| KA3 | 3 | 0 | 0 | 1 | 0 | 1 | 1 | 0 | 0 | 0 | 0 | 0 | 0 | 6 | 34^th^ |
| KA6 | 0 | 0 | 1 | 0 | 0 | 1 | 1 | 0 | 0 | 3 | 0 | 0 | 0 | 6 | 35^th^ |
| KA24 | 0 | 0 | 0 | 1 | 1 | 0 | 1 | 3 | 0 | 0 | 0 | 0 | 0 | 6 | 36^th^ |
| KA40 | 1 | 1 | 1 | 1 | 0 | 1 | 1 | 0 | 0 | 0 | 0 | 0 | 0 | 6 | 37^th^ |
| KA50 | 0 | 0 | 0 | 1 | 1 | 1 | 1 | 0 | 2 | 0 | 0 | 0 | 0 | 6 | 38^th^ |
| KA68 | 0 | 0 | 0 | 1 | 1 | 1 | 1 | 0 | 2 | 0 | 0 | 0 | 0 | 6 | 39^th^ |
| KA71 | 0 | 0 | 0 | 1 | 1 | 1 | 1 | 0 | 2 | 0 | 0 | 0 | 0 | 6 | 40^th^ |
| MA10 | 3 | 0 | 1 | 0 | 0 | 1 | 1 | 0 | 0 | 0 | 0 | 0 | 0 | 6 | 41^st^ |
| MA13 | 3 | 0 | 1 | 0 | 0 | 1 | 1 | 0 | 0 | 0 | 0 | 0 | 0 | 6 | 42^nd^ |
| MA37 | 1 | 0 | 1 | 1 | 0 | 1 | 1 | 0 | 1 | 0 | 0 | 0 | 0 | 6 | 43^rd^ |
| KA47 | 3 | 0 | 1 | 0 | 0 | 1 | 0 | 0 | 0 | 0 | 0 | 0 | 0 | 5 | 44^th^ |
| KA55 | 0 | 1 | 1 | 1 | 1 | 1 | 0 | 0 | 0 | 0 | 0 | 0 | 0 | 5 | 45^th^ |
| MA43 | 1 | 1 | 1 | 1 | 0 | 1 | 0 | 0 | 0 | 0 | 0 | 0 | 0 | 5 | 46^th^ |
| K86 | 1 | 0 | 1 | 1 | 1 | 1 | 0 | 0 | 0 | 0 | 0 | 0 | 0 | 5 | 47^th^ |
| KA38 | 1 | 1 | 1 | 0 | 0 | 1 | 1 | 0 | 0 | 0 | 0 | 0 | 0 | 5 | 48^th^ |
| KA51 | 1 | 0 | 1 | 1 | 0 | 1 | 1 | 0 | 0 | 0 | 0 | 0 | 0 | 5 | 49^th^ |
| MA8 | 3 | 0 | 1 | 0 | 0 | 0 | 1 | 0 | 0 | 0 | 0 | 0 | 0 | 5 | 50^th^ |
| MA9 | 0 | 0 | 1 | 0 | 1 | 1 | 1 | 0 | 0 | 0 | 0 | 0 | 1 | 5 | 51^st^ |
| MA63 | 1 | 0 | 1 | 1 | 0 | 1 | 1 | 0 | 0 | 0 | 0 | 0 | 0 | 5 | 52^nd^ |
| MA69 | 2 | 0 | 1 | 0 | 0 | 1 | 1 | 0 | 0 | 0 | 0 | 0 | 0 | 5 | 53^rd^ |
| M34 | 1 | 0 | 1 | 1 | 1 | 0 | 0 | 0 | 0 | 0 | 0 | 0 | 0 | 4 | 54^th^ |
| MA54 | 2 | 1 | 1 | 0 | 0 | 0 | 0 | 0 | 0 | 0 | 0 | 0 | 0 | 4 | 55^th^ |
| MA68 | 3 | 0 | 1 | 0 | 0 | 0 | 0 | 0 | 0 | 0 | 0 | 0 | 0 | 4 | 56^th^ |
| K27 | 1 | 0 | 1 | 1 | 1 | 0 | 0 | 0 | 0 | 0 | 0 | 0 | 0 | 4 | 57^th^ |
| K78 | 1 | 0 | 1 | 1 | 1 | 0 | 0 | 0 | 0 | 0 | 0 | 0 | 0 | 4 | 58^th^ |
| K91 | 1 | 0 | 1 | 0 | 1 | 1 | 0 | 0 | 0 | 0 | 0 | 0 | 0 | 4 | 59^th^ |
| K92 | 1 | 0 | 1 | 0 | 1 | 1 | 0 | 0 | 0 | 0 | 0 | 0 | 0 | 4 | 60^th^ |
| M18 | 1 | 1 | 1 | 0 | 1 | 0 | 0 | 0 | 0 | 0 | 0 | 0 | 0 | 4 | 61^st^ |
| KA45 | 1 | 0 | 1 | 0 | 0 | 1 | 1 | 0 | 0 | 0 | 0 | 0 | 0 | 4 | 62^nd^ |
| KA52 | 1 | 0 | 1 | 0 | 0 | 1 | 1 | 0 | 0 | 0 | 0 | 0 | 0 | 4 | 63^rd^ |
| KA61 | 0 | 2 | 0 | 1 | 0 | 0 | 1 | 0 | 0 | 0 | 0 | 0 | 0 | 4 | 64^th^ |
| MA11 | 1 | 0 | 1 | 0 | 0 | 1 | 1 | 0 | 0 | 0 | 0 | 0 | 0 | 4 | 65^th^ |
| MA66 | 1 | 0 | 1 | 0 | 0 | 1 | 1 | 0 | 0 | 0 | 0 | 0 | 0 | 4 | 66^th^ |
| MA71 | 1 | 0 | 1 | 0 | 0 | 1 | 1 | 0 | 0 | 0 | 0 | 0 | 0 | 4 | 67^th^ |
| KA32 | 0 | 1 | 1 | 0 | 0 | 1 | 0 | 0 | 0 | 0 | 0 | 0 | 0 | 3 | 68^th^ |
| KA54 | 0 | 1 | 0 | 0 | 1 | 1 | 0 | 0 | 0 | 0 | 0 | 0 | 0 | 3 | 69^th^ |
| KA89 | 0 | 2 | 0 | 1 | 0 | 0 | 0 | 0 | 0 | 0 | 0 | 0 | 0 | 3 | 70^th^ |
| MA26 | 1 | 0 | 1 | 0 | 0 | 1 | 0 | 0 | 0 | 0 | 0 | 0 | 0 | 3 | 71^st^ |
| MA48 | 0 | 0 | 1 | 0 | 0 | 1 | 0 | 0 | 0 | 0 | 0 | 0 | 1 | 3 | 72^nd^ |
| M15 | 1 | 0 | 1 | 0 | 1 | 0 | 0 | 0 | 0 | 0 | 0 | 0 | 0 | 3 | 73^rd^ |
| M36 | 1 | 0 | 1 | 0 | 1 | 0 | 0 | 0 | 0 | 0 | 0 | 0 | 0 | 3 | 74^th^ |
| KA28 | 0 | 0 | 0 | 1 | 1 | 0 | 1 | 0 | 0 | 0 | 0 | 0 | 0 | 3 | 75^th^ |
| KA30 | 0 | 0 | 0 | 0 | 1 | 1 | 1 | 0 | 0 | 0 | 0 | 0 | 0 | 3 | 76^th^ |
| KA82 | 0 | 1 | 0 | 0 | 0 | 1 | 1 | 0 | 0 | 0 | 0 | 0 | 0 | 3 | 77^th^ |
| MA70 | 1 | 0 | 1 | 0 | 0 | 0 | 1 | 0 | 0 | 0 | 0 | 0 | 0 | 3 | 78^th^ |
| KA87 | 0 | 0 | 0 | 1 | 1 | 0 | 0 | 0 | 0 | 0 | 0 | 0 | 0 | 2 | 79^th^ |
| KA88 | 0 | 0 | 0 | 0 | 1 | 1 | 0 | 0 | 0 | 0 | 0 | 0 | 0 | 2 | 80^th^ |
| MA57 | 0 | 0 | 1 | 0 | 0 | 1 | 0 | 0 | 0 | 0 | 0 | 0 | 0 | 2 | 81^st^ |
| K33 | 0 | 0 | 1 | 1 | 0 | 0 | 0 | 0 | 0 | 0 | 0 | 0 | 0 | 2 | 82^nd^ |
| K34 | 0 | 0 | 1 | 1 | 0 | 0 | 0 | 0 | 0 | 0 | 0 | 0 | 0 | 2 | 83^rd^ |
| KA5 | 0 | 0 | 1 | 0 | 0 | 0 | 1 | 0 | 0 | 0 | 0 | 0 | 0 | 2 | 84^th^ |
| KA16 | 0 | 0 | 1 | 0 | 0 | 0 | 1 | 0 | 0 | 0 | 0 | 0 | 0 | 2 | 85^th^ |
| KA76 | 0 | 0 | 0 | 1 | 0 | 0 | 1 | 0 | 0 | 0 | 0 | 0 | 0 | 2 | 86^th^ |
| KA25 | 0 | 0 | 1 | 0 | 0 | 0 | 0 | 0 | 0 | 0 | 0 | 0 | 0 | 1 | 87^th^ |
| KA85 | 0 | 0 | 1 | 0 | 0 | 0 | 0 | 0 | 0 | 0 | 0 | 0 | 0 | 1 | 88^th^ |

IAA^a^, Indole acetic acid production; PS^b^, Phosphate solubilization; AM^c^, Ammonia production; NF^d^, Nitrogen fixation; ACC^e^, ACC deaminase production; Sid^f^, Siderophore production; Chi^g^, Chitinase production; Fo^h^, *F. oxysporum* (MTCC 284); Ph^i^, *P. hypobrunnea* (ITCC 4141); Rs^j^, *R. solani* (MTCC 4633); Ns^k^, *N. sphaerica* (KJ767520); Pl^l^, *P. lamaensis* (ITCC 292); Ev^m^, *E. vexans* (ITCC 938).
