# Supplementary material for: Characterization and selection of endophytic actinobacteria for growth and disease management of Tea (Camellia sinensis L.)
Source: Front Plant Sci. 2022 Nov 9;13:989794. doi: 10.3389/fpls.2022.989794 (PMC9681920; doi:10.3389/fpls.2022.989794)
Supplement: Supplementary file 4 [file Table_4.docx]

**TABLE S4.** Evaluation of induced systemic resistance by *Streptomyces* sp. KA12.

| **Sample** | | **Number of tomato seeds per score category (n)** | | | | |
| --- | --- | --- | --- | --- | --- | --- |
|  |  | **No seed germination**  **(Score= n x 0)** | **Seed infection**  **(Score= n x 1)** | **Healthy seed showing germination**  **(Score= n x 2)** | **Germination (%)** | **Total score** |
| Non-bacterized seeds | Sterile water | 11 | None | 4 | 26.6 ± 0.4 | 8 |
|  | Culture media | 12 | None | 3 | 20 ± 0.28 | 6 |
|  | 0.1% CMC | 11 | None | 4 | 26.6 ± 0.63 | 8 |
| *F. oxysporum* treated seeds | | 14 | 14 | 1 | 6.6 ± 0.81 | 16 |
| Bacterized seeds | KA12 | 3 | None | 12 | 80 ± 0.43 | 24 |
|  | KA12 and *F. oxysporum* | 4 | 4 | 11 | 73.33 ± 0.25 | 22 |
