# Supplementary figures and images for: Characterization and selection of endophytic actinobacteria for growth and disease management of Tea (Camellia sinensis L.)
Source: Front Plant Sci. 2022 Nov 9;13:989794. doi: 10.3389/fpls.2022.989794 (PMC9681920; doi:10.3389/fpls.2022.989794)

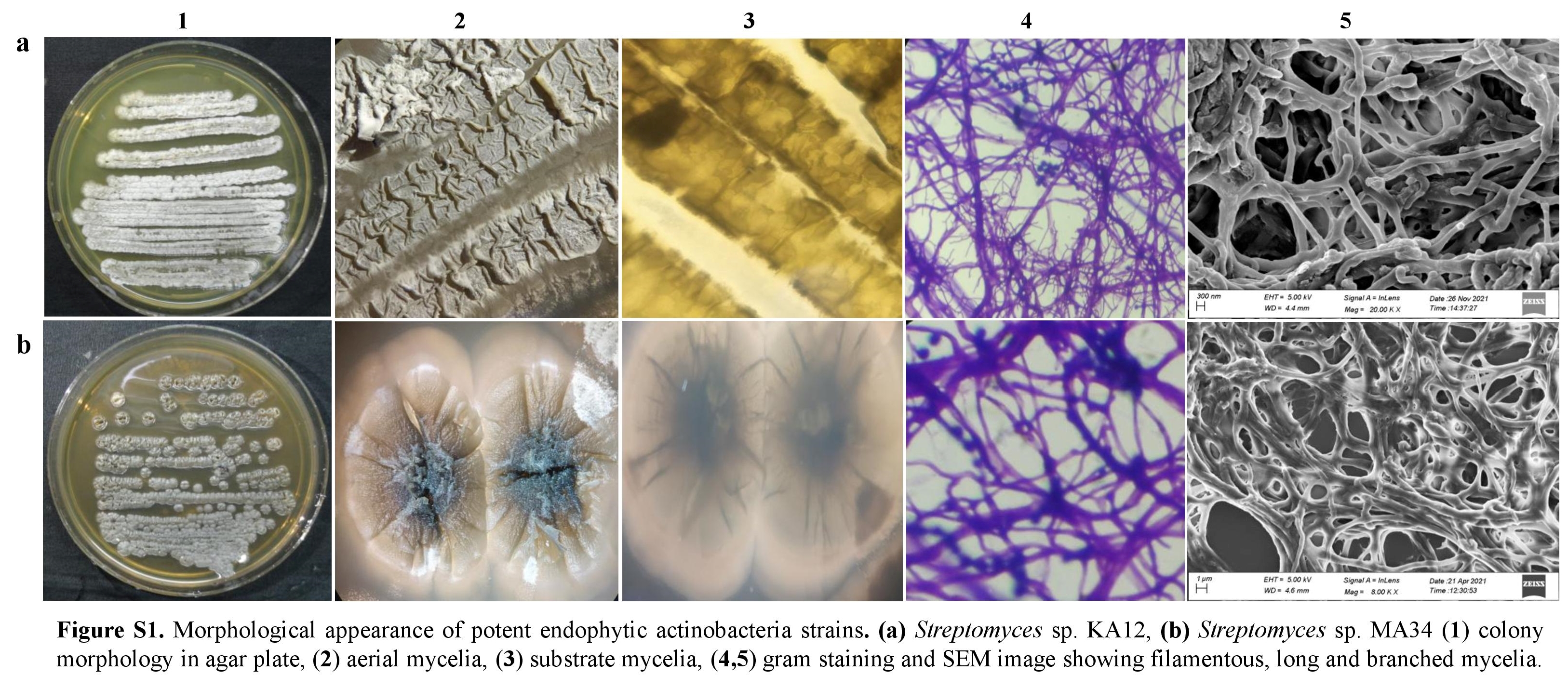

Supplement: Supplementary file 5 [file Image_1.jpeg]

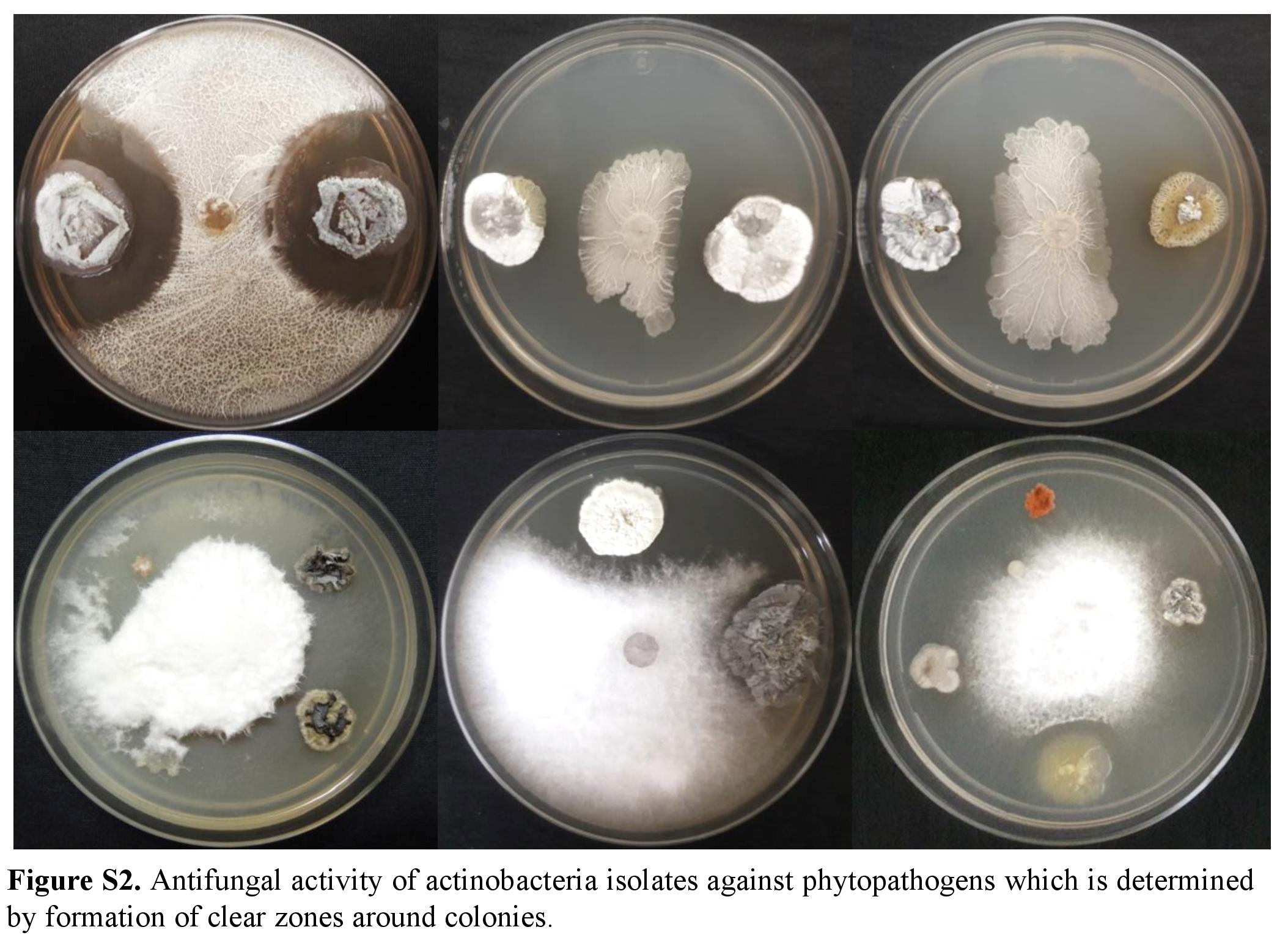

Supplement: Supplementary file 6 [file Image_2.jpeg]

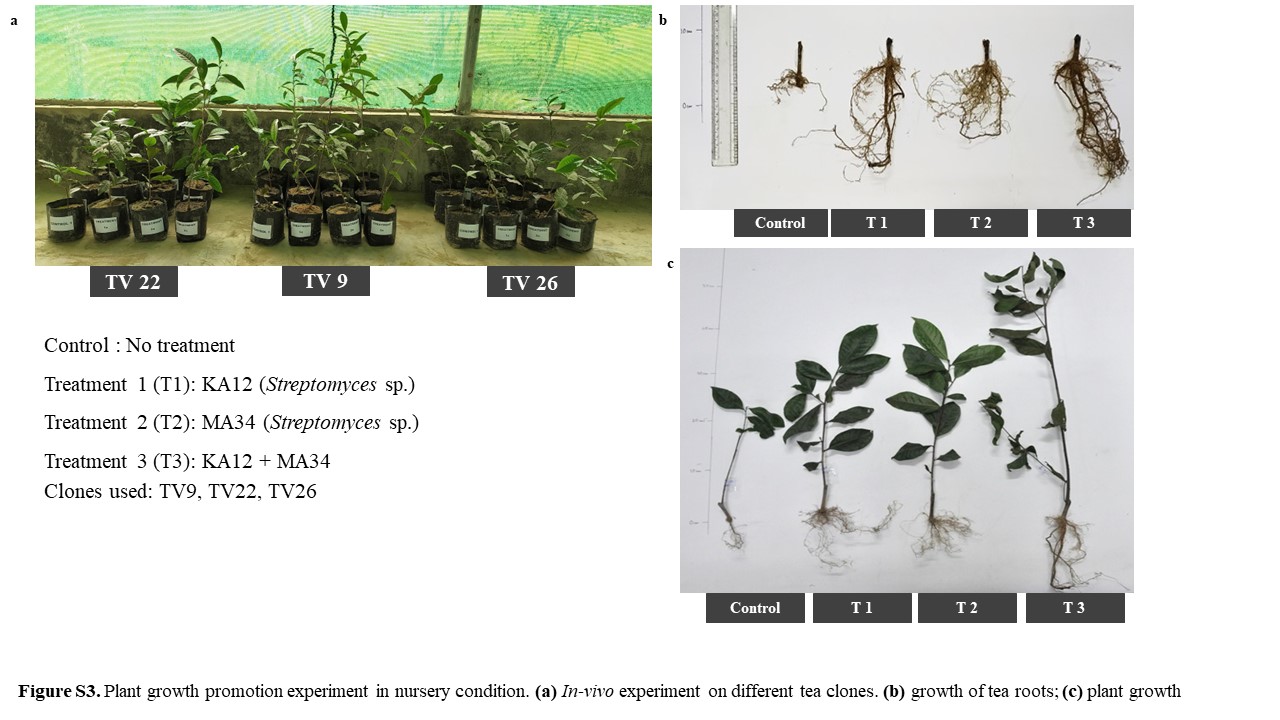

Supplement: Supplementary file 7 [file Image_3.jpeg]

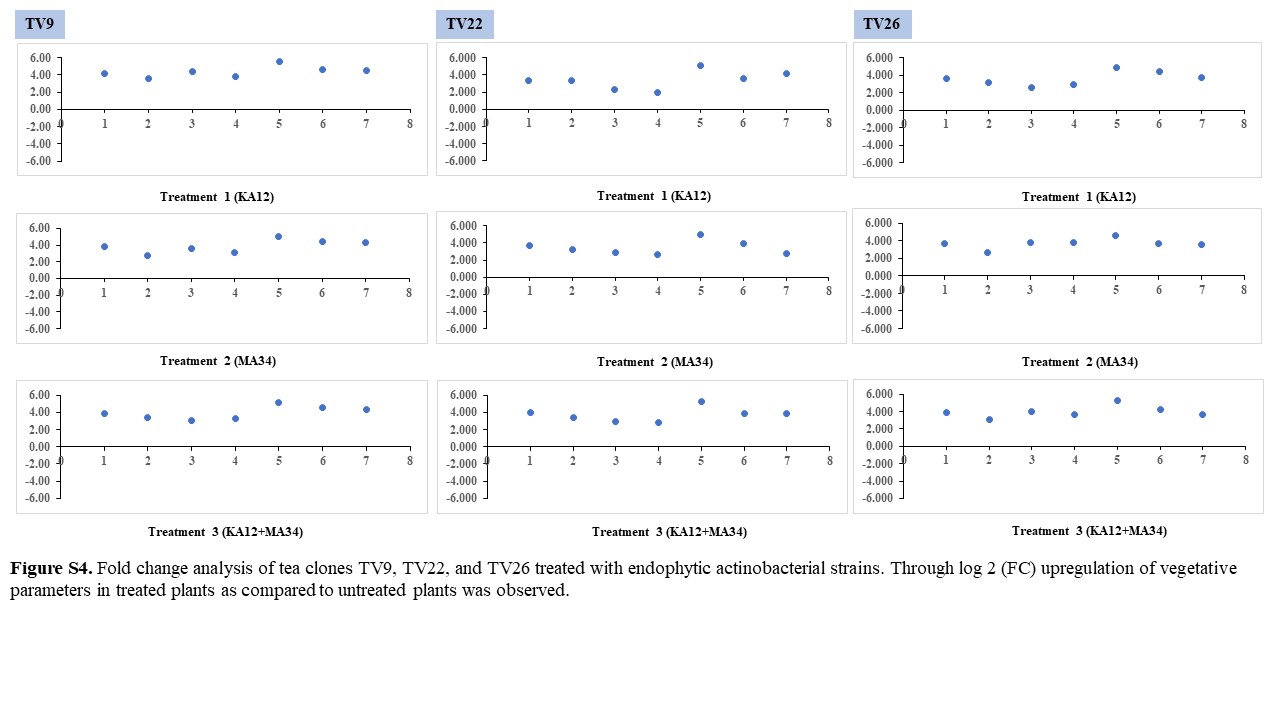

Supplement: Supplementary file 8 [file Image_4.jpeg]
